# FGFR2b in Gastric Cancer: Translating a Therapeutic Target into a Reliable Biomarker

**DOI:** 10.3390/cancers18121863

**Published:** 2026-06-06

**Authors:** Catalin-Bogdan Satala, Gabriela Gurău, Gabriela Patrichi, Alina-Mihaela Gurau, Roxana-Cristina Mehedinti, Andy Radu Leibovici, Daniela Mihalache

**Affiliations:** 1 Medical and Pharmaceutical Research Center, Faculty of Medicine and Pharmacy, “Dunarea de Jos” University of Galati, 800008 Galati, Romania; catalin.satala@ugal.ro (C.-B.S.); gabriela.gurau@ugal.ro (G.G.); roxana.mehedinti@ugal.ro (R.-C.M.); daniela.mihalache@ugal.ro (D.M.); 2 Department of Pathology, Clinical County Emergency Hospital Braila, 810325 Braila, Romania; 3 “Sf. Ioan” Clinical Emergency Pediatric Hospital, 800487 Galati, Romania; 4 The Doctoral School of Medicine and Pharmacy, “George Emil Palade” University of Medicine, Pharmacy, Science and Technology, 540142 Targu Mures, Romania; 5 The School for Doctoral Studies in Biomedical Sciences, “Dunarea de Jos” University of Galati, 800008 Galati, Romania; alina.gurau@ugal.ro

**Keywords:** FGFR2b, fibroblast growth factor receptor 2b, gastric cancer, gastroesophageal junction cancer, biomarker heterogeneity, targeted therapy

## Abstract

Fibroblast growth factor receptor 2b (FGFR2b) has become an increasingly important therapeutic target in gastric and gastroesophageal junction cancer, particularly with the clinical development of FGFR2b-directed antibody therapy. However, its translation into routine treatment selection is not straightforward. FGFR2b is usually assessed as a protein biomarker by immunohistochemistry, and a positive result may reflect different biological situations depending on staining intensity, percentage of positive tumor cells, sample type and spatial distribution. In addition, FGFR2b protein expression, FGFR2 amplification, transcript-level activity and true pathway dependency are related but not interchangeable. This review examines FGFR2b-positive gastric cancer from the perspective of biomarker reliability rather than target presence alone. We discuss the biological basis of FGFR2b targeting, the reasons for variability in reported positivity rates, the implications of intratumoral and inter-lesion heterogeneity, the current clinical evidence for FGFR2b-directed and broader FGFR-targeted approaches, and the emerging challenges of safety, resistance and treatment sequencing. Particular attention is given to the gap between detecting FGFR2b and identifying tumors in which this target is sufficiently expressed, representative and biologically relevant to guide therapy. We also consider how FGFR2b should be interpreted alongside HER2, CLDN18.2, immune biomarkers and other receptor tyrosine kinase alterations. As FGFR2b-directed strategies move forward, their success will depend not only on drug efficacy, but also on standardized testing, careful reporting, and selective reassessment when disease biology changes. FGFR2b therefore offers a useful model for how protein biomarkers can be developed in gastric cancer: not as isolated positive-or-negative labels, but as clinically interpreted variables within a changing therapeutic landscape.

## Simple Summary

Fibroblast growth factor receptor 2b (FGFR2b) is one of the most promising new therapeutic targets in gastric and gastroesophageal junction cancer, but identifying the patients who may truly benefit is not simple. A positive FGFR2b test does not always mean the same thing: expression may be strong or weak, diffuse or focal, present in one tumor site but absent in another, or be biologically less important than other active pathways. This review looks at FGFR2b not only as a target, but as a biomarker that must be interpreted carefully. We discuss how FGFR2b is tested, why reported positivity rates differ, how tumor heterogeneity may affect treatment response, and how FGFR2b-directed therapy fits into the broader landscape of gastric cancer biomarkers. The central message is that FGFR2b should not be treated as a simple positive-or-negative label, but as a clinically interpreted variable.

## 1. Introduction: FGFR2b as a Stress Test for Precision Oncology in Gastric Cancer

Gastric and gastroesophageal junction adenocarcinomas are increasingly managed within a biomarker-driven therapeutic framework. In recent years, treatment selection has expanded beyond conventional clinicopathological parameters to include molecular and immunohistochemical markers such as HER2, programmed death-ligand 1 combined positive score, mismatch repair or microsatellite instability status, and claudin 18.2 expression [1,2,3,4]. This transition has improved the ability to identify patients for targeted and immune-based approaches, but it has also exposed a central limitation of precision oncology in gastric cancer: biomarker positivity does not necessarily imply durable therapeutic benefit. Spatial heterogeneity, temporal biomarker instability, incomplete pathway dependency, and adaptive resistance can all limit the predictive value of a single baseline test [5].

Fibroblast growth factor receptor 2b has emerged in this context as a clinically relevant but biologically complex biomarker [6]. FGFR2b is an epithelial splice isoform of FGFR2, and its overexpression or related FGFR2 alterations have been associated with a subgroup of gastric and gastroesophageal junction cancers that may be amenable to FGFR2b-directed therapy [6,7]. Unlike broad genomic alterations alone, FGFR2b is commonly assessed at the protein level, most often by immunohistochemistry, which introduces practical questions regarding assay selection, staining thresholds, tumor sampling, and interpretation of heterogeneous expression. These issues are particularly relevant in gastric cancer, where biomarker expression may vary not only within the primary tumor but also between the primary site and metastatic lesions [7,8].

The clinical development of FGFR2b-directed therapy has provided proof of concept for this target. The randomized phase II FIGHT trial evaluated bemarituzumab, a monoclonal antibody directed against FGFR2b, in combination with mFOLFOX6 in advanced gastric or gastroesophageal junction adenocarcinoma [9]. Final analysis reported continued evidence of clinical activity and manageable safety in FGFR2-selected, HER2-negative disease, with a more pronounced treatment effect observed in patients whose tumors showed FGFR2b overexpression in at least 10% of tumor cells using 2+/3+ staining criteria [10]. Subsequent phase III development has further reinforced the clinical relevance of this pathway. FORTITUDE-101 is designed to compare bemarituzumab plus mFOLFOX6 with placebo plus mFOLFOX6 in previously untreated advanced gastric or gastroesophageal junction cancer with FGFR2b overexpression [11]. FORTITUDE-102 is evaluating bemarituzumab in combination with chemotherapy and nivolumab compared with chemotherapy and nivolumab alone in a similar FGFR2b-overexpressing first-line population [12].

However, the emergence of FGFR2b as a therapeutic target should not lead to a simplified view of FGFR2b-positive disease. Several unresolved questions remain. First, FGFR2b positivity is not defined uniformly across studies, and reported prevalence varies according to assay, cut-off, sample type, and disease setting. Second, FGFR2b protein overexpression, FGFR2 amplification, FGFR2 mRNA expression, and true FGFR2 pathway dependency are related but not interchangeable biological states. Third, spatial heterogeneity may create both diagnostic uncertainty and a biological basis for incomplete response. Fourth, resistance to FGFR2b-directed therapy may arise through several non-mutually exclusive mechanisms, including pre-existing FGFR2b-low subclones, treatment-induced antigen modulation, bypass signaling through parallel receptor tyrosine kinase pathways, phenotypic plasticity, and tumor microenvironment-mediated protection [10,13].

For these reasons, FGFR2b may be best understood not as a static positive-or-negative biomarker, but as a dynamic therapeutic variable. Its clinical utility will likely depend on how accurately it is defined at baseline, how well heterogeneity is captured across tumor sites, how resistance is monitored during treatment, and how FGFR2b-directed therapy is integrated with other biomarker-driven options such as HER2-, CLDN18.2-, VEGF/VEGFR-, and immune checkpoint-directed strategies. This is especially important as the gastric cancer treatment landscape becomes increasingly crowded with overlapping molecular targets and potential treatment sequences [14,15,16,17,18].

This review examines FGFR2b-positive gastric cancer from this dynamic biomarker perspective. Rather than providing a general overview of the FGFR family, we focus on the specific translational questions that will determine the clinical value of FGFR2b-directed therapy: the biological meaning of FGFR2b positivity, the causes of variability in detection, the impact of spatial heterogeneity, the current clinical evidence, emerging mechanisms of resistance, interactions with the tumor microenvironment, integration with other actionable biomarkers, and future strategies for longitudinal monitoring. By framing FGFR2b as a model of dynamic biomarker implementation, this review aims to clarify both the opportunities and the unresolved challenges associated with targeting FGFR2b in gastric and gastroesophageal junction cancer.

## 2. Literature Search and Review Approach

This article was designed as a narrative review with a translational and clinically oriented focus. The aim was not to perform a formal systematic review or meta-analysis, but to synthesize the available biological, pathological and clinical evidence relevant to FGFR2b as a therapeutic biomarker in gastric and gastroesophageal junction cancer.

Relevant publications were identified through searches of PubMed/MEDLINE and ClinicalTrials.gov, supplemented by manual screening of reference lists from key articles. The search focused on studies addressing FGFR2 or FGFR2b biology, FGFR2b protein expression, FGFR2 amplification, immunohistochemical testing, spatial heterogeneity, FGFR-directed therapies, bemarituzumab, resistance mechanisms and biomarker implementation in gastric or gastroesophageal junction adenocarcinoma. Search terms included combinations of “gastric cancer”, “gastroesophageal junction cancer”, “FGFR2”, “FGFR2b”, “FGFR2-IIIb”, “FGFR2 amplification”, “bemarituzumab”, “FIGHT trial”, “FORTITUDE”, “immunohistochemistry”, “heterogeneity”, “biomarker”, and “resistance”.

Priority was given to peer-reviewed original studies, clinical trials, biomarker analyses, translational studies, and relevant reviews. Clinical trial registry entries were used to describe ongoing studies when mature peer-reviewed results were not yet available. Publications were selected based on their relevance to the biological interpretation, detection, clinical targeting and implementation of FGFR2b in gastric cancer. Because this was a narrative review, no formal risk-of-bias assessment, quantitative evidence grading or meta-analytic synthesis was performed.

The evidence was organized thematically to emphasize the distinction between FGFR2b target detection and therapeutic relevance, including protein expression, genomic alteration, transcript-level activity, spatial heterogeneity, clinical efficacy, safety, resistance and integration with other biomarkers. This structure was chosen to support a biomarker-centered interpretation of FGFR2b-positive gastric cancer rather than a purely chronological summary of the literature.

## 3. The FGFR2b Axis: From Epithelial Isoform to Oncogenic Dependency

Fibroblast growth factor receptor 2 is a receptor tyrosine kinase involved in the regulation of cell proliferation, differentiation, survival, migration, and tissue repair. Its biological effects depend not only on receptor expression, but also on alternative splicing, ligand availability, cellular context, and downstream signaling activity. Among the FGFR2 isoforms, FGFR2b, also referred to as FGFR2-IIIb, is predominantly expressed in epithelial tissues, whereas FGFR2c, or FGFR2-IIIc, is more commonly associated with mesenchymal compartments [19]. This epithelial–mesenchymal distinction is biologically relevant in gastric cancer because it links FGFR2 signaling to tumor differentiation state, plasticity, and potentially to therapeutic vulnerability [20].

The structural difference between FGFR2b and FGFR2c results from alternative splicing of the third immunoglobulin-like domain of the receptor. This splicing event modifies ligand-binding specificity and influences the cellular programs activated after receptor engagement. FGFR2b preferentially binds a subset of epithelial-associated fibroblast growth factors, while FGFR2c has a different ligand-binding profile and is more closely linked to mesenchymal signaling contexts. Therefore, FGFR2 isoform identity is not merely a nomenclature issue; it may reflect distinct biological states within the tumor and its surrounding microenvironment [19,20].

In gastric cancer, aberrant activation of the FGFR2 pathway may occur through several mechanisms, including FGFR2 amplification, increased FGFR2b protein expression, enhanced ligand-dependent signaling, or broader dysregulation of receptor tyrosine kinase networks. Once activated, FGFR2 signaling can engage canonical downstream pathways such as RAS–RAF–MAPK, PI3K–AKT, PLCγ, and STAT-related signaling. These pathways contribute to proliferation, survival, motility, angiogenic signaling, and resistance to cellular stress. Importantly, these downstream cascades are not unique to FGFR2b; they overlap with signaling programs activated by other receptor tyrosine kinases, including HER2, EGFR, MET, and VEGFR-related pathways. This overlap is one reason why FGFR2b expression alone may not always translate into exclusive FGFR2b dependency, and why earlier evaluations of FGFR2 targeting in gastric cancer emphasized both its therapeutic promise and the risk of overinterpreting FGFR2 alterations without robust biomarker selection [21].

The concept of oncogenic dependency is central to understanding FGFR2b as a therapeutic target. A tumor may express FGFR2b at the protein level, but this does not necessarily mean that tumor growth and survival are primarily driven by FGFR2b signaling. Conversely, tumors with FGFR2 amplification may show stronger pathway activation, but amplification and protein overexpression are not fully interchangeable biological states [22]. This distinction is particularly important in gastric cancer, where molecular heterogeneity and parallel oncogenic drivers can complicate the interpretation of a single biomarker result. In practical terms, FGFR2b positivity should be considered a potential indicator of targetability, while true FGFR2 pathway addiction requires a broader biological context.

FGFR2 isoform biology may also be connected to tumor phenotype. Earlier gastric cancer studies suggested that FGFR2-IIIb and FGFR2-IIIc expression are not equivalent in their clinicopathological associations. FGFR2-IIIb expression appears more closely aligned with epithelial tumor biology, whereas FGFR2-IIIc expression has been associated with more mesenchymal features and may carry distinct prognostic implications [20]. More recent data further support the relevance of FGFR2-IIIc as a marker linked to mesenchymal transition and unfavorable tumor behavior in gastric and gastroesophageal junction cancers [23]. These observations do not prove that isoform switching is a universal mechanism of treatment resistance, but they do suggest that FGFR2 isoform composition may influence tumor phenotype and therapeutic sensitivity.

A clinically important implication is that FGFR2b-directed therapy targets a specific protein isoform rather than all forms of FGFR2 pathway activation. This distinction separates FGFR2b-directed monoclonal antibody therapy from broader small-molecule FGFR inhibition. Antibody-based FGFR2b targeting depends on sufficient cell-surface expression of the FGFR2b protein, adequate tumor penetration, and maintenance of the target during therapy [11,12]. By contrast, small-molecule FGFR inhibitors are generally designed to inhibit kinase activity across one or more FGFR family members and may be more directly relevant to tumors with kinase-dependent FGFR alterations [24,25]. These therapeutic classes therefore raise different questions regarding patient selection, resistance mechanisms, and toxicity.

From a translational perspective, the key biological question is not simply whether FGFR2b is present, but whether FGFR2b expression identifies a therapeutically meaningful state. This requires distinguishing among four related but non-identical concepts: FGFR2b protein expression, FGFR2 genomic alteration, active FGFR signaling, and functional tumor dependency. The separation of these concepts is essential for interpreting trial results, refining biomarker thresholds, and designing future resistance-monitoring strategies.

Overall, FGFR2b represents a biologically plausible and clinically relevant target in gastric cancer because it connects epithelial receptor expression with oncogenic signaling and therapeutic accessibility. However, its role as a biomarker is inherently layered. FGFR2b expression may indicate target presence, but target presence alone does not guarantee pathway dependency, uniform expression across tumor sites, or durable therapeutic sensitivity. This biological complexity makes the definition of FGFR2b positivity clinically consequential rather than purely descriptive.

## 4. Defining FGFR2b Positivity: Protein, Gene, Transcript and Functional State

The increasing clinical interest in FGFR2b-directed therapy has made the definition of “FGFR2b-positive” gastric cancer a central translational issue. At first glance, FGFR2b positivity may appear to be a straightforward biomarker category. In practice, however, it can refer to several related but biologically distinct states: FGFR2b protein overexpression, FGFR2 gene amplification, increased FGFR2 transcript expression, isoform-specific expression, or functional dependence on FGFR2-driven signaling. These categories overlap, but they are not interchangeable. Recognizing this distinction is essential for interpreting clinical trial results, estimating prevalence, selecting patients for therapy, and designing future resistance-monitoring strategies [22].

The most clinically developed approach for identifying FGFR2b-positive tumors is immunohistochemical assessment of FGFR2b protein expression [8,26]. This method evaluates the presence and intensity of FGFR2b staining in tumor tissue, usually emphasizing membranous expression because antibody-based targeting depends on cell-surface availability of the receptor. In clinical studies of bemarituzumab, FGFR2b selection has been based on immunohistochemistry, with positivity commonly defined by moderate-to-strong membranous staining. The phase II FIGHT trial enrolled patients with FGFR2b-positive, HER2-negative gastric or gastroesophageal junction adenocarcinoma defined by 2+/3+ membranous staining by immunohistochemistry [9]. Earlier publication of the randomized FIGHT study also established the clinical rationale for selecting patients by FGFR2b expression in this setting [10].

Protein expression is clinically intuitive because it directly reflects the presence of the therapeutic target at the tumor-cell surface. However, immunohistochemistry does not measure pathway activity directly. A tumor with detectable FGFR2b staining may not necessarily depend on FGFR2b signaling for growth or survival, particularly if parallel receptor tyrosine kinase pathways or downstream alterations dominate the tumor biology. Conversely, a tumor may harbor FGFR2 amplification but show heterogeneous or limited protein expression depending on sampling, transcriptional regulation, or technical factors. Therefore, FGFR2b immunohistochemistry is best viewed as a target-detection tool rather than a complete measure of biological dependency.

FGFR2 amplification represents a second, related biomarker layer. Amplification may increase receptor density and support oncogenic signaling, and it has historically provided a rationale for FGFR-targeted approaches in gastric cancer. Earlier studies reported FGFR2 amplification in a small subset of gastric cancers and linked it to the development of FGFR-directed therapeutic strategies [27]. Other analyses showed that FGFR2 gene amplification and FGFR2 protein overexpression are associated but not perfectly concordant, emphasizing the need for accurate screening methods and careful interpretation of biomarker results [22]. This partial concordance is important: amplification may suggest pathway relevance, but antibody-based FGFR2b therapy still requires sufficient expression of the FGFR2b isoform at the protein level.

A third layer is transcript-level assessment. FGFR2 mRNA expression can provide information about transcriptional activation and, when isoform-specific methods are used, may help distinguish FGFR2b/IIIb from FGFR2c/IIIc. This is biologically meaningful because isoform identity may correspond to different epithelial or mesenchymal tumor states. Nevertheless, mRNA-based testing is not yet as clinically standardized as immunohistochemistry for patient selection in FGFR2b-directed therapy. Differences in tissue processing, RNA quality, assay platform, and analytic threshold can all affect interpretation. For this reason, transcript-level analysis is currently better suited for translational studies, mechanistic research, and exploratory biomarker development than for routine stand-alone selection [20].

The fourth and most complex layer is functional FGFR2 pathway dependency. This refers to whether tumor growth, survival, invasion, or treatment tolerance is meaningfully driven by FGFR2 signaling. Functional dependency cannot be inferred from a single biomarker in all cases. It is shaped by receptor abundance, ligand availability, receptor activation, downstream pathway wiring, co-occurring molecular alterations, and microenvironmental signals. This distinction is clinically relevant because a therapy can only produce durable benefit if target expression is accompanied by sufficient biological reliance on that target. In other words, FGFR2b positivity identifies a candidate therapeutic vulnerability, whereas FGFR2 dependency defines the biological strength of that vulnerability [28]. Although *FGFR2* amplification and FGFR2b protein overexpression may coexist, they should be interpreted as partially overlapping rather than equivalent biomarker states. The available evidence suggests that concordance depends strongly on assay platform, scoring threshold, cohort composition and tissue sampling. For example, studies enriched for strong FGFR2b expression may show high concordance with *FGFR2* amplification, whereas broader IHC definitions may identify a larger group of tumors with detectable membranous FGFR2b expression but no demonstrable high-level amplification. Conversely, an amplification-positive result does not automatically establish that the FGFR2b isoform is uniformly and sufficiently expressed at the tumor-cell surface for antibody-based targeting. Therefore, the practical clinical question is not simply whether FGFR2 is altered, but which biomarker layer is being measured and which therapeutic modality is being considered. This distinction is particularly relevant because FGFR2b-directed monoclonal antibodies require accessible membranous FGFR2b protein, whereas small-molecule FGFR inhibitors are more closely linked to kinase-pathway activation and genomic or pathway-level FGFR alterations. A structured comparison of these biomarker layers is provided in Table 1.

The distinction between FGFR2b protein expression and *FGFR2* amplification is clinically important because these markers define overlapping, but not identical, patient populations. In FORTITUDE-101 prescreening, the apparent prevalence of FGFR2b positivity changed from 37.8% with any 2+/3+ membranous staining to 16.2% when a ≥10% tumor-cell threshold was required. By contrast, FGFR2 amplification is generally reported in a smaller subset of gastric cancers. Earlier work showed that FGFR2 protein overexpression can predict FGFR2 gene amplification, supporting biological overlap, but this does not make IHC and amplification interchangeable. The practical consequence is that IHC, FISH/NGS, ctDNA, RNA-based assays and functional assays should be interpreted as complementary layers, each answering a different clinical question.

A useful way to conceptualize FGFR2b status is therefore to separate “target presence” from “target dependence.” Tumors with high FGFR2b protein expression and FGFR2 amplification may be more likely to show meaningful pathway reliance, although this relationship still requires clinical validation across treatment settings. Tumors with high protein expression but no amplification may remain targetable by antibody-based therapy, but the degree of FGFR2-driven biology may vary. Tumors with amplification but low or heterogeneous FGFR2b staining raise a different problem: the genomic alteration may be present, but the surface target required for FGFR2b-directed antibody therapy may be insufficiently expressed or unevenly distributed. Finally, tumors lacking both FGFR2b expression and FGFR2 amplification are less likely to benefit from FGFR2b-directed strategies, although they may still harbor other actionable pathways [10,20].

This layered interpretation also helps clarify why reported biomarker-positive populations differ across studies. A cohort defined by any FGFR2b membranous staining will not be equivalent to a cohort defined by a higher percentage of tumor cells with 2+/3+ staining. Similarly, a population selected by FGFR2 amplification is not identical to one selected by FGFR2b protein overexpression. These definitions may enrich for overlapping but biologically distinct patient groups. As a result, cross-study comparisons should account for whether the biomarker criterion reflects protein expression, copy-number alteration, transcript abundance, or a composite definition.

For clinical implementation, this distinction has practical consequences. If FGFR2b is used as a therapeutic biomarker, pathology reports should ideally describe not only positivity or negativity, but also staining intensity, percentage of positive tumor cells, membranous localization, and, when available, the relationship to FGFR2 amplification or broader molecular profiling. Such detail may become increasingly important as treatment decisions involve multiple actionable biomarkers and as clinicians consider therapy sequencing after progression. A binary biomarker label may be insufficient in a disease where the target can be heterogeneous, context-dependent, and potentially altered by prior therapy [13].

**Figure 1:**
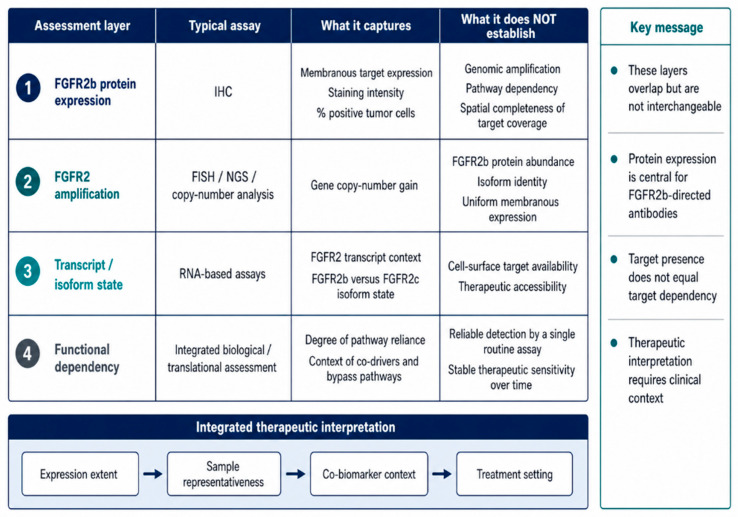
FGFR2b biomarker interpretation: non-equivalent layers of assessment. FGFR2b positivity should be interpreted beyond a single assay result. The model highlights the distinction between detecting a therapeutic target and establishing its clinical relevance for FGFR2b-directed treatment (the figure is intended as a conceptual framework; readers should refer to the corresponding sections of the main text and to the supporting tables for the evidence base, assay-specific limitations, and clinical interpretation underlying the relationships illustrated here).

In summary, FGFR2b positivity should be understood as a multidimensional biomarker state. Protein expression identifies the physical target for FGFR2b-directed antibody therapy; FGFR2 amplification may indicate genomic activation of the pathway; transcript-level analysis may refine isoform biology; and functional dependency determines whether the pathway is likely to represent a clinically meaningful vulnerability. This distinction is summarized in Figure 1, which frames FGFR2b interpretation as a layered process moving from target detection to therapeutic relevance, while emphasizing that protein expression, genomic alteration, transcript or isoform state, and functional dependency should not be treated as interchangeable variables.

Once these biomarker layers are separated, variability in reported FGFR2b prevalence becomes easier to interpret as a consequence of assay choice, cut-off definition and tissue sampling.

## 5. The Prevalence Paradox: Why Reported FGFR2b Rates Vary

Reported rates of FGFR2b positivity in gastric and gastroesophageal junction cancer vary substantially across studies. This variability should not be viewed simply as disagreement between datasets, but rather as a consequence of how FGFR2b is defined, measured and sampled. Unlike a purely genomic alteration with a relatively fixed detection framework, FGFR2b is most often assessed as a protein biomarker by immunohistochemistry, where staining intensity, membranous localization, percentage of positive tumor cells and tissue representativeness all influence classification. As a result, FGFR2b prevalence is not a single universal number. It is a context-dependent estimate shaped by assay design, cut-off selection, specimen type and the biological layer being interrogated [7,13,20].

This point is important because prevalence estimates influence more than epidemiological description. They affect screening strategies, trial feasibility, biomarker enrichment, pathology workload and expectations regarding the number of patients who may be eligible for FGFR2b-directed therapy. Therefore, understanding why reported rates differ is essential before interpreting FGFR2b as a routine therapeutic biomarker.

### 5.1. Cut-Off-Dependent Prevalence

The most direct explanation for variation across studies is the cut-off used to define positivity. Immunohistochemistry captures FGFR2b expression along a continuum, but clinical studies must convert this continuum into categorical groups. A broad definition that accepts moderate-to-strong staining in any proportion of tumor cells will identify more cases than a stricter definition requiring the same intensity in a minimum percentage of malignant cells.

In prescreening analysis from the FORTITUDE-101, FGFR2b protein overexpression defined as 2+/3+ membranous staining in any percentage of tumor cells was reported in 37.8% of samples, whereas 2+/3+ staining in at least 10% of tumor cells was observed in 16.2% of samples [13]. These values are not contradictory; they represent different thresholds applied to the same biomarker concept.

This cut-off dependence has direct biological and clinical implications. A tumor with focal FGFR2b expression may not be equivalent to one in which a broad fraction of cells shows strong membranous staining. For antibody-based therapy, target distribution matters because the therapeutic antigen must be present on the surface of enough tumor cells to support meaningful engagement. A permissive threshold may improve sensitivity and reduce the risk of excluding patients with potentially targetable disease, but it may also include tumors in which FGFR2b expression is limited or spatially restricted. A more stringent threshold may enrich for tumors with greater target availability, but it reduces the eligible population.

Thus, the optimal definition of FGFR2b positivity should not be determined by prevalence alone. It should ideally reflect the relationship between expression level, reproducibility of scoring, clinical benefit and treatment tolerability. This is especially relevant for borderline cases, where weak, focal or heterogeneous staining may be interpreted differently depending on the assay, scoring algorithm and reader experience.

### 5.2. Sample-Dependent Prevalence

Cut-off selection is only one part of the problem. Even when the same threshold is used, the type of tissue analyzed can influence FGFR2b positivity rates. FGFR2b status may be assessed on surgical resections, endoscopic biopsies, archival primary tumors or metastatic samples, and these sources do not necessarily provide equivalent information.

Larger specimens may capture a wider range of intratumoral expression, whereas small biopsies may under-sample positive areas or overrepresent a highly positive focus. A metastatic biopsy may better reflect the disease burden being treated in the advanced setting, but metastatic tissue is not always available and may be limited in quantity or quality. In this context, a recent study reporting a low FGFR2b positivity rate of 4.1% in gastric cancer is informative because the authors linked the low detection rate to heterogeneous expression and sampling limitations [29]. Rather than suggesting that one prevalence estimate is correct and another is wrong, such differences indicate that FGFR2b detection is highly sensitive to how much tissue is examined, which lesion is tested and how positivity is scored.

The timing of tissue acquisition adds another layer of complexity. Archival pretreatment tissue may not fully represent the biomarker status at the moment a treatment decision is made, particularly in patients who have received intervening systemic therapy. Conversely, newly obtained biopsies may better reflect current disease biology but are often small and may not capture the full spatial complexity of the tumor. This issue is not unique to FGFR2b, but it is especially relevant for a biomarker whose expression may be focal or unevenly distributed [30,31].

For clinical purposes, the practical question is therefore not only whether a tumor has ever shown FGFR2b expression, but whether the available sample reliably reflects the targetable disease at the time therapy is being considered. The same sampling issue also points to a deeper biological problem: FGFR2b expression may be unevenly distributed within and across tumor sites.

### 5.3. Biomarker Layer Influences Prevalence Estimates

A further reason for divergent prevalence estimates is that different studies do not always measure the same biomarker layer. Some define cases by FGFR2b protein expression, others by FGFR2 amplification, copy-number gain or RNA expression. These categories are related, but they do not identify identical populations [20,23].

This distinction was introduced in the previous section, but its practical consequence is important: frequencies derived from IHC, FISH, NGS or transcriptomic assays should not be compared as if they measured the same biological state. An IHC-defined FGFR2b-positive cohort may include tumors without high-level FGFR2 amplification, while an amplification-defined cohort may include cases with variable FGFR2b protein expression. Similarly, transcript-level estimates may not correspond directly to membrane target availability [13,20,23].

The therapeutic modality also determines which biomarker layer is most relevant. For an FGFR2b-directed antibody strategy, accessible cell-surface protein expression is essential [11,12]. For broader FGFR kinase inhibition, genomic or pathway-level evidence may carry different significance [24,25]. Therefore, prevalence estimates must be interpreted in relation to both the assay used and the type of treatment being considered.

This perspective helps avoid a common interpretive error: treating “FGFR2b-positive,” “FGFR2-amplified” and “FGFR2-overexpressing” as interchangeable terms. They may overlap, but they are not synonymous. Clear terminology is therefore necessary when comparing studies, designing trials and translating results into clinical practice.

### 5.4. Practical Implications for Clinical Implementation and Trial Design

The prevalence paradox has direct consequences for implementation. Laboratories need validated immunohistochemical procedures and reproducible scoring systems. Clinicians need pathology reports that provide enough detail to interpret borderline or heterogeneous staining. Trial designers need to decide whether they aim for broad inclusion or biomarker enrichment. Each of these decisions changes the apparent size and biological composition of the FGFR2b-positive population.

A binary positive-or-negative report may be insufficient for FGFR2b. More informative reporting should ideally include staining intensity, percentage of positive tumor cells, membranous localization, sample type and whether the tested tissue came from a primary or metastatic site. In selected cases, complementary molecular testing for FGFR2 amplification or broader receptor tyrosine kinase alterations may help contextualize ambiguous findings, although such tests should not be treated as direct substitutes for FGFR2b protein assessment when an FGFR2b-directed antibody strategy is being considered.

The same issue applies to clinical trials. A broad eligibility definition may facilitate enrolment and capture a larger biologically diverse population, whereas a stricter definition may enrich for tumors more likely to maintain the therapeutic target. These two approaches answer different clinical questions. Broad inclusion asks whether any detectable FGFR2b expression can identify a treatable group; enriched selection asks whether stronger or more extensive expression better predicts benefit. Future studies will need to clarify which definition best balances access, predictive value and treatment burden.

In summary, the reported frequency of FGFR2b positivity depends on cut-off selection, tissue sampling, assay modality and the biological layer being measured. This variability is not merely a methodological limitation; it is a central feature of FGFR2b as a clinical biomarker. Before FGFR2b-directed therapy can be fully integrated into routine precision oncology, the field must define not only who is positive, but also how much expression is sufficient, which sample should be trusted and whether the detected target reflects a therapeutically relevant disease state. Even after a tumor is classified as FGFR2b-positive, the clinical meaning of that result depends on how the target is distributed across the disease burden.

## 6. Spatial Heterogeneity: The Hidden Architecture of FGFR2b-Positive Disease

FGFR2b positivity is usually reported as a property of a tumor sample, but therapeutic response depends on the biology of the whole disease burden. This distinction is particularly important in gastric and gastroesophageal junction cancer, where molecular and protein-level heterogeneity can occur within the primary tumor, between different regions of the same lesion, and across metastatic sites, for multiple markers [5,30,32]. As discussed in the previous section, sampling influences the apparent prevalence of FGFR2b positivity. The present section extends that issue from detection to biology: spatial heterogeneity is not only a diagnostic challenge, but also a potential determinant of primary resistance, incomplete response, and disease progression under treatment pressure.

### 6.1. Intratumoral Heterogeneity

Intratumoral heterogeneity refers to the coexistence of biologically distinct tumor cell populations within the same lesion [5]. In the context of FGFR2b, this may mean that some regions show strong membranous expression, whereas others show weak, focal, or absent staining. Such uneven expression can complicate both biomarker interpretation and therapeutic targeting. A tumor classified as FGFR2b-positive on the basis of one sampled region may still contain clinically relevant FGFR2b-low or FGFR2b-negative compartments [29].

This concept is especially relevant for immunohistochemical biomarkers because staining patterns are spatial by nature. A single percentage or positivity label may compress a complex distribution into a simplified category. For example, two tumors may both meet a predefined cut-off, yet differ substantially in the distribution of positive cells: one may show diffuse moderate-to-strong expression, while another may show intense expression restricted to a limited area. These patterns may not be equivalent for an antibody-based treatment, where therapeutic activity depends on target accessibility across malignant cells.

Recent data support the importance of heterogeneous FGFR2b expression in gastric cancer. Lee et al. reported a low FGFR2b positivity rate and emphasized that heterogeneous expression may limit reliable detection using limited tissue material [29]. This finding does not only affect prevalence estimates; it also raises the possibility that some tumors classified as positive may contain mixed target-positive and target-negative compartments, while some tumors classified as negative may have unsampled positive regions.

A practical implication is that the percentage of FGFR2b-positive cells may be as important as staining intensity. Strong expression in a small fraction of cells may indicate target presence, but not necessarily broad target coverage. Conversely, moderate expression across a large fraction of tumor cells may represent a different therapeutic scenario. Therefore, intratumoral distribution should be considered part of FGFR2b interpretation, rather than an incidental observation.

### 6.2. Inter-Lesion Heterogeneity

Beyond variation within a single tumor mass, FGFR2b expression may differ between disease sites. In advanced gastric cancer, treatment decisions are often based on tissue obtained from the primary tumor, while the clinically relevant disease burden may include lymph node, liver, peritoneal, or other metastatic deposits [33,34,35]. If FGFR2b expression differs between these sites, a single biopsy may not accurately reflect the biology of all lesions requiring treatment [8].

Inter-lesion heterogeneity has several possible consequences. If the primary tumor is FGFR2b-positive but metastatic lesions are partly or entirely FGFR2b-negative, an FGFR2b-directed therapy may exert uneven pressure across disease sites. Some lesions may respond, while others may remain stable or progress. Conversely, if the primary tumor is negative but a metastatic site is positive, testing only the primary lesion could exclude a patient who might otherwise have targetable disease. These scenarios remain incompletely characterized for FGFR2b, but they are biologically plausible and clinically important.

**Figure 2:**
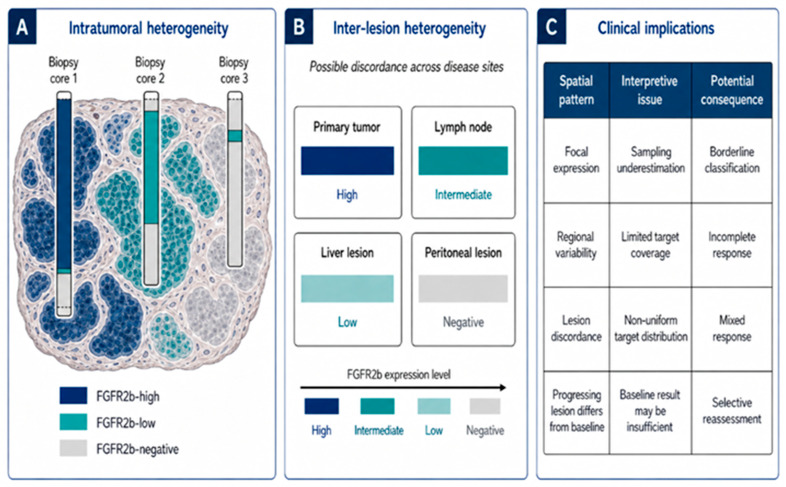
Spatial heterogeneity of FGFR2b expression and its clinical implications. (**A**) Different biopsy cores may capture different proportions of FGFR2b-high, FGFR2b-low and FGFR2b-negative regions within the same tumor. (**B**) Possible inter-lesion discordance, where primary and metastatic sites may show different relative levels of FGFR2b expression. (**C**) Potential clinical implications, including sampling uncertainty, limited target coverage, mixed response patterns and the need for selective reassessment during disease evolution (The figure illustrates potential sampling and interpretation scenarios rather than fixed biological categories; direct evidence for site-specific FGFR2b expression patterns remains limited and should be interpreted according to anatomical site, tissue adequacy and assay threshold; readers should refer to the corresponding sections of the main text and to the supporting tables for the evidence base, assay-specific limitations, and clinical interpretation underlying the relationships illustrated here).

The issue is not unique to FGFR2b; several gastric cancer biomarkers show spatial variability [5,32,36]. However, FGFR2b may be especially sensitive to this problem because the therapeutic target is assessed by protein expression, and target distribution is directly relevant for antibody-based treatment. For this reason, pathology reporting should ideally specify whether FGFR2b status was determined from the primary tumor, a metastatic lesion, or archival tissue, and whether the sample was representative enough to support confident interpretation. The practical implications of intratumoral and inter-lesion variability are summarized in Figure 2, which illustrates how spatial patterns of FGFR2b expression may influence sampling, biomarker classification and lesion-level interpretation.

### 6.3. Peritoneal Disease as a Special Setting

Peritoneal dissemination deserves particular attention in FGFR2b-positive gastric cancer because it combines three clinically relevant problems: difficult tissue access, spatial biomarker heterogeneity and a distinct immune-stromal microenvironment. Peritoneal disease is common in advanced gastric cancer, is often challenging to assess by conventional imaging, and may be represented by small-volume implants, ascites-associated tumor cells or fibrotic/stromal-rich deposits. These features can influence both the reliability of FGFR2b testing and the interpretation of treatment response.

The key evidence-based point is that peritoneal metastases should not currently be assumed to be uniformly FGFR2b-high or FGFR2b-low. Published data remain limited, and the available evidence does not establish a reproducible direction of FGFR2b expression change in peritoneal deposits compared with hematogenous metastases such as liver lesions. Rather, the most defensible conclusion is that peritoneal dissemination may show spatial heterogeneity and site-level discordance. A recent analysis comparing FGFR2b expression in primary gastric cancer and peritoneal dissemination reported heterogeneity and discordance between tumor compartments, supporting the view that evaluation of a single anatomical site may not fully capture FGFR2b status in advanced disease [37,38,39].

This distinction is clinically important. If FGFR2b expression differs between the primary tumor and peritoneal implants, treatment selection based only on archival primary tumor tissue may be incomplete. Conversely, a negative or focal result from a small peritoneal biopsy may reflect limited tissue volume, low tumor cellularity, stromal dilution or sampling bias rather than true absence of the target across the peritoneal disease burden. Therefore, when peritoneal tissue is available, the anatomical origin, tumor cellularity and adequacy of the specimen should be explicitly considered in FGFR2b interpretation.

The biological links between FGFR2b and peritoneal disease also remain incompletely defined. Peritoneal metastases develop within a microenvironment enriched in mesothelial interactions, cancer-associated fibroblasts, extracellular matrix remodeling, immune suppression and ascitic fluid-derived growth factors. These factors may influence FGFR ligand availability, receptor signaling, epithelial–mesenchymal plasticity, drug penetration and resistance. However, direct evidence connecting these microenvironmental features to altered FGFR2b expression or bemarituzumab sensitivity in peritoneal metastases is still limited. Thus, the peritoneal microenvironment should be discussed as a plausible modifier of FGFR2b-directed therapy rather than as a proven mechanism of resistance [40,41].

Future FGFR2b studies should include dedicated analyses of peritoneal disease, ideally with paired sampling of primary tumors, peritoneal implants and hematogenous metastases. Such studies should determine whether FGFR2b expression is preserved, lost, enriched or spatially restricted in peritoneal dissemination; whether stromal-rich or low-cellularity samples affect scoring reproducibility; and whether peritoneal-dominant progression is associated with loss of FGFR2b expression, bypass signaling or microenvironment-mediated resistance. Until such data mature, peritoneal disease should be regarded as an evidence-limited but clinically important setting in which FGFR2b testing requires particular caution.

### 6.4. Spatial Heterogeneity as Primary Resistance

Spatial heterogeneity can create the appearance of primary resistance even when the biomarker is technically present. If a tumor contains a mixture of FGFR2b-high and FGFR2b-low populations before treatment, the FGFR2b-high compartment may be vulnerable to targeted therapy, while the FGFR2b-low compartment may persist. Clinically, this could manifest as partial response, mixed response, early progression in selected lesions, or limited durability of benefit [29].

This mechanism differs from acquired resistance. In primary resistance driven by spatial heterogeneity, resistant cell populations already exist before treatment begins. Therapy does not necessarily create them; it reveals their clinical relevance by suppressing the target-positive fraction while sparing less dependent or target-negative components. This is why the extent and distribution of FGFR2b expression may matter as much as simple positivity [42].

This idea also provides a biological explanation for why more stringent FGFR2b cut-offs may enrich for benefit. A higher proportion of FGFR2b-positive tumor cells may indicate broader target coverage and fewer pre-existing target-negative compartments. However, this remains a hypothesis that requires prospective validation. It should not be assumed that higher expression always predicts better response in every context, because therapeutic activity may also depend on pathway dependency, co-drivers, microenvironmental influences, and treatment combination.

For this reason, future trials should not only classify tumors as positive or negative, but should also examine whether the spatial pattern of staining correlates with response. Digital pathology and image-based heterogeneity metrics may eventually help convert spatial patterns into reproducible quantitative variables.

### 6.5. Spatial Heterogeneity as a Basis for Acquired Resistance

The same spatial architecture may also contribute to acquired resistance. Under treatment pressure, FGFR2b-positive clones may be reduced, while FGFR2b-low or FGFR2b-negative clones may expand. In this setting, progression does not require the emergence of a completely new resistant mechanism; it may reflect selection of pre-existing subclones that were underrepresented at baseline. This model is well established in principle for targeted therapy and is highly relevant to FGFR2b because expression heterogeneity may be present before treatment [42].

At progression, several spatially informed questions become important. Has FGFR2b expression decreased in progressing lesions? Do residual tumors retain the target but activate bypass pathways? Are different metastases progressing through different mechanisms? Is resistance uniform across the disease burden, or does it vary by anatomical site? These questions cannot be answered reliably by baseline testing alone.

Re-biopsy at progression, when feasible, may therefore be particularly informative in FGFR2b-positive disease. However, tissue-based reassessment should be interpreted alongside its limitations: a single progression biopsy may again sample only one resistant compartment. Complementary approaches such as circulating tumor DNA (ctDNA) may help capture genomic evolution across multiple disease sites, particularly when tissue re-biopsy is difficult or when progression is anatomically heterogeneous. In advanced gastric cancer, ctDNA analysis has been shown to detect *FGFR2* amplification and concurrent genomic alterations associated with FGFR inhibitor activity, supporting its value as a non-invasive tool for identifying FGFR2-driven disease and tracking co-alterations over time [43]. In the FGFR2b setting, ctDNA may be especially useful for detecting persistence or loss of *FGFR2* amplification, emergence of bypass alterations involving parallel RTK pathways, or downstream genomic events that could contribute to acquired resistance. However, ctDNA cannot directly measure FGFR2b protein expression, membranous target availability or spatial distribution. Thus, ctDNA and tissue reassessment answer complementary, not identical, questions: ctDNA is best suited for monitoring genomic evolution, whereas tissue remains necessary to assess the antibody-accessible FGFR2b protein target.

Spatial heterogeneity also has implications for combination therapy. If resistance arises partly from pre-existing FGFR2b-negative compartments, combining FGFR2b-directed therapy with chemotherapy, immune checkpoint inhibition, anti-angiogenic therapy, or another targeted strategy may help suppress a broader tumor population. However, rational combinations should be guided by biological evidence rather than by the assumption that more treatment automatically overcomes heterogeneity [10].

### 6.6. Implications for Pathology and Translational Research

Recognizing spatial heterogeneity changes how FGFR2b testing should be interpreted. From a research perspective, FGFR2b heterogeneity should be studied prospectively rather than treated as a technical inconvenience. Paired primary and metastatic samples, multiple biopsies from the same patient, digital image analysis, spatial transcriptomics, and correlation with lesion-level radiologic response could all help clarify how target distribution affects treatment outcome. Importantly, such studies should distinguish between heterogeneity of FGFR2b protein expression, heterogeneity of FGFR2 amplification, and heterogeneity of downstream pathway activation.

In summary, spatial heterogeneity is central to the clinical interpretation of FGFR2b-positive gastric cancer. It can influence prevalence estimates, patient selection, primary sensitivity, mixed response, and the emergence of resistance. Although the previous section addressed how sampling affects reported positivity rates, the present section highlights a deeper point: uneven FGFR2b expression may be part of the biological architecture of the disease itself. These considerations are central to interpreting the clinical evidence for FGFR2b-directed therapy, where treatment effect is inseparable from how the biomarker-selected population is defined.

## 7. Clinical Evidence: From Proof-of-Concept to Phase III Development

The clinical development of FGFR2b-directed therapy has moved FGFR2b from a biologically plausible target to a clinically testable biomarker in gastric and gastroesophageal junction cancer. The evidence base is still developing, but it has already shaped several important principles. FGFR2b-directed therapy has been evaluated most prominently in HER2-negative advanced disease, patient selection has relied primarily on FGFR2b protein expression by immunohistochemistry, and the interpretation of benefit depends closely on the biomarker threshold used to define the treated population. At the same time, the clinical literature includes several different FGFR-directed approaches, and these should not be treated as interchangeable. Monoclonal antibodies directed against FGFR2b, broader FGFR kinase inhibitors, and FGFR2-directed antibody–drug conjugates differ in target biology, biomarker requirements, resistance mechanisms, and toxicity profiles.

### 7.1. Bemarituzumab and the Rationale for FGFR2b-Directed Antibody Therapy

Bemarituzumab is the most clinically advanced FGFR2b-directed agent in gastric and gastroesophageal junction cancer. It is a monoclonal antibody designed to bind the FGFR2b isoform at the tumor-cell surface. This mechanism makes FGFR2b protein expression central to patient selection, because the therapeutic target must be accessible on the cell membrane. This point distinguishes FGFR2b-directed antibody therapy from small-molecule FGFR inhibition, where the relevant biomarker may be more closely linked to kinase pathway activation, FGFR2 amplification, or other FGFR pathway alterations [44].

The rationale for bemarituzumab is based on selective targeting of a tumor-associated epithelial isoform rather than broad inhibition of the FGFR family. This selectivity is clinically attractive because it may allow pathway-directed therapy in a biomarker-defined subset while avoiding some limitations of less selective FGFR blockade [9,44]. Mechanistically, bemarituzumab has been described as inhibiting ligand binding to FGFR2b and as having potential antibody-dependent cellular cytotoxic activity [44]. In advanced gastric cancer, this provides a rationale for combination with cytotoxic chemotherapy, where receptor targeting and chemotherapy-mediated tumor cell killing may act through complementary mechanisms.

However, the same mechanism also creates specific biomarker requirements. For an antibody directed against FGFR2b, the presence, intensity, and distribution of membranous staining are clinically meaningful. As discussed in earlier sections, FGFR2b positivity should not be interpreted only as target presence; it should also be considered in relation to the proportion of tumor cells expressing the target and the extent to which the tumor may depend on FGFR2b-related signaling. The clinical development of bemarituzumab therefore tests both a therapeutic strategy and a biomarker-selection model [13].

### 7.2. The FIGHT Trial: Proof of Concept for FGFR2b-Selected Therapy

The randomized phase II FIGHT trial provided the key proof of concept for FGFR2b-directed therapy in advanced gastric and gastroesophageal junction adenocarcinoma. The study evaluated bemarituzumab plus mFOLFOX6 versus placebo plus mFOLFOX6 as first-line treatment in patients with HER2-negative advanced disease selected for FGFR2b overexpression and/or FGFR2 amplification. The initial report showed encouraging clinical activity with the addition of bemarituzumab to chemotherapy and established FGFR2b-selected disease as a relevant investigational subgroup [9]. The final analysis of FIGHT further supported the clinical activity of this approach. Bemarituzumab plus mFOLFOX6 continued to show evidence of benefit compared with chemotherapy alone in the FGFR2b-selected population, with a safety profile considered manageable in the context of advanced disease treatment. Importantly, the final analysis also suggested that patients whose tumors had FGFR2b overexpression in at least 10% of tumor cells derived a more pronounced benefit than the broader biomarker-selected population [10].

This finding is highly relevant for the biomarker framework of this review. It supports the idea that FGFR2b expression is not simply a qualitative marker, but a quantitative and spatially influenced variable. A tumor with limited focal expression may not have the same therapeutic profile as a tumor with more extensive membranous expression. The FIGHT data therefore do more than support bemarituzumab activity; they reinforce the need to define FGFR2b positivity in a way that reflects therapeutic relevance. At the same time, FIGHT should be interpreted as a phase II study [9,10]. Its major contribution was to establish clinical plausibility, guide biomarker refinement, and justify confirmatory phase III development. It was not designed to resolve all questions regarding optimal cut-off, spatial heterogeneity, sequencing, or resistance. Those questions remain central to the next stage of FGFR2b clinical development.

### 7.3. Regional Analyses and Patient-Reported Outcomes

Additional analyses of FIGHT have provided useful information on regional consistency and patient-centered outcomes. In the East Asian subgroup, bemarituzumab plus mFOLFOX6 showed findings generally aligned with the overall direction of the study, supporting further evaluation of FGFR2b-directed therapy across populations in which gastric cancer is common [45]. These analyses are valuable because treatment patterns, molecular epidemiology, and clinical outcomes in gastric cancer can vary across regions. Nevertheless, subgroup results should be interpreted in the context of sample size and exploratory intent.

Health-related quality-of-life data are also important for the interpretation of FGFR2b-directed therapy. In advanced gastric cancer, additional treatment intensity is clinically meaningful only if it provides benefit without unacceptable deterioration in patient well-being. The FIGHT quality-of-life analysis suggested that adding bemarituzumab to mFOLFOX6 did not produce a major worsening of health-related quality of life compared with chemotherapy alone during the evaluated period [46]. This complements traditional endpoints such as progression-free survival and overall survival by addressing tolerability from the patient perspective.

These analyses do not replace the primary efficacy findings, but they strengthen the clinical context in which FGFR2b-directed therapy is being developed. They suggest that the approach can be evaluated not only in terms of tumor control, but also in relation to patient experience, regional applicability, and feasibility of combination with standard chemotherapy.

### 7.4. Phase III Development: Confirmatory Trials and Evolving Clinical Questions

Following the proof-of-concept provided by FIGHT, phase III development has been designed to determine whether FGFR2b-directed therapy provides clinically meaningful benefit in a definitive first-line setting. This transition is particularly important because phase III studies are not only testing a therapeutic regimen; they are also testing whether FGFR2b protein expression can serve as a reproducible and clinically useful selection biomarker across larger, more diverse populations.

FORTITUDE-101 is a randomized, double-blind, placebo-controlled phase III study evaluating bemarituzumab plus mFOLFOX6 versus placebo plus mFOLFOX6 in previously untreated, unresectable locally advanced or metastatic gastric or gastroesophageal junction adenocarcinoma with FGFR2b overexpression and HER2-negative status. The study is designed to assess whether adding FGFR2b-directed therapy to chemotherapy improves clinical outcomes in a prospectively selected population. Its design is relevant because it places FGFR2b testing directly into the first-line treatment setting, where therapeutic decisions are increasingly shaped by multiple biomarkers. The importance of FORTITUDE-101 extends beyond the primary efficacy endpoints. It may help clarify whether the degree of FGFR2b expression influences treatment benefit, whether a more restrictive immunohistochemical threshold better enriches for clinically meaningful response, and whether FGFR2b scoring can be implemented reproducibly across a large screening program. Publicly presented phase III data have reported an overall survival benefit at the primary analysis, while longer follow-up and full peer-reviewed datasets will be important for interpreting the durability of the effect, biomarker-defined subgroups and the clinical magnitude of benefit. From a translational perspective, the most informative data will be those linking clinical outcomes to expression level, staining distribution, sample type, safety, subsequent therapies, and molecular correlates of progression [11].

FORTITUDE-102 addresses a related but distinct question. This phase Ib/III study evaluates bemarituzumab in combination with mFOLFOX6 and nivolumab compared with mFOLFOX6 and nivolumab alone in previously untreated advanced gastric or gastroesophageal junction cancer with FGFR2b overexpression. The rationale is clinically relevant because immune checkpoint inhibition has become an important component of first-line treatment for selected patients with advanced gastric cancer. Therefore, FORTITUDE-102 does not simply test whether FGFR2b-directed therapy can be added to chemotherapy; it asks whether FGFR2b targeting can be integrated into a chemoimmunotherapy backbone [12].

This distinction is important. If FGFR2b-directed therapy is developed only in combination with chemotherapy, its clinical position may remain relatively narrow. If it can be combined safely and effectively with immune checkpoint inhibition, its role may become more closely aligned with contemporary first-line treatment strategies. At the same time, triplet therapy raises additional questions regarding toxicity, biomarker hierarchy, patient selection, and the biological relationship between FGFR2b signaling and antitumor immunity. These questions require careful interpretation rather than assuming that treatment intensification is automatically beneficial.

Overall, the phase III program should be viewed as an evolving source of evidence that will refine the place of FGFR2b-directed therapy. Its value lies not only in confirming efficacy, but also in defining how FGFR2b should be measured, what threshold is clinically meaningful, and how FGFR2b-directed therapy should be combined with other active first-line components. Despite the strong rationale for confirmatory phase III development, several interpretive issues remain important. The FIGHT trial established proof of concept, but as a randomized phase II study it cannot fully resolve the optimal FGFR2b threshold, the relative contribution of FGFR2b expression versus FGFR2 amplification, or the distinction between predictive and prognostic biomarker value. High FGFR2b expression may identify tumors more likely to benefit from bemarituzumab, but it may also define a biologically distinct subgroup with aggressive clinical behavior in some datasets. Therefore, randomized phase III data and detailed biomarker-outcome correlations are needed to determine whether FGFR2b functions primarily as a treatment-predictive biomarker, a prognostic marker, or both.

The IHC criteria used for patient selection should also be interpreted as clinically pragmatic but still empirically evolving. The observation that benefit appeared more pronounced in tumors with FGFR2b overexpression in at least 10% of tumor cells supports the importance of expression extent, but it does not prove that this threshold is biologically optimal across all settings. Cut-off selection influences prevalence, enrichment, assay reproducibility and access to therapy. A permissive threshold may capture more patients with target-positive disease, whereas a more stringent threshold may enrich for tumors with broader target coverage and possibly stronger FGFR2b dependence.

Finally, clinical implementation may be challenging even if phase III efficacy is confirmed. FGFR2b testing must be integrated into an already crowded first-line biomarker workflow that includes HER2, PD-L1, mismatch repair or microsatellite instability status and CLDN18.2. Tissue availability, archival versus fresh sampling, primary versus metastatic site selection, heterogeneous staining and turnaround time may all affect real-world eligibility. These challenges mean that the clinical value of FGFR2b-directed therapy will depend not only on trial efficacy, but also on reproducible testing, appropriate cut-off selection and careful interpretation of discordant biomarker results.

### 7.5. Later-Line and Combination Strategies

Although first-line therapy has been the main focus of bemarituzumab development, later-line and combination strategies are also clinically relevant. One such approach is the evaluation of bemarituzumab with ramucirumab and paclitaxel in FGFR2b-positive advanced gastric or gastroesophageal junction cancer. This strategy is of interest because ramucirumab plus paclitaxel is an established later-line regimen, and adding FGFR2b targeting explores whether the pathway remains therapeutically relevant after prior treatment exposure [47].

The later-line setting raises different biomarker questions from first-line therapy. FGFR2b expression measured at diagnosis may not fully reflect the status of the target after chemotherapy, targeted therapy, or immune checkpoint inhibition. If FGFR2b expression changes over time, then contemporary tissue or correlative biomarker analyses may be especially important in later-line trials. This is not a minor procedural point; it relates directly to whether FGFR2b is a stable therapeutic marker or a dynamic feature of evolving disease.

Combination strategies also need a clear biological rationale. FGFR2b-directed therapy may be combined with chemotherapy to broaden cytotoxic activity, with anti-angiogenic therapy to address vascular and stromal escape mechanisms, or with immune checkpoint inhibition to integrate targeted and immune-based treatment [47]. However, the value of any combination depends on whether the added agent improves outcomes beyond what would be expected from the backbone regimen, and whether the toxicity remains acceptable. For this reason, combinations should be interpreted through both efficacy and biomarker lenses.

### 7.6. Lessons from FGFR Kinase Inhibition in Gastric Cancer

Small-molecule FGFR inhibitors are relevant to the broader FGFR2 therapeutic landscape in gastric and gastroesophageal junction cancer, but they should not be conflated with FGFR2b-directed antibody therapy. Their therapeutic logic is different: they inhibit kinase activity across one or more FGFR family members and are generally more closely linked to FGFR2 amplification, FGFR fusions or pathway-level FGFR activation than to FGFR2b membranous protein expression. By contrast, bemarituzumab depends on sufficient cell-surface FGFR2b expression. Therefore, FGFR2 amplification, FGFR pathway activation and FGFR2b IHC positivity should not be treated as interchangeable selection strategies.

Clinical experience with broader FGFR inhibition in gastric cancer has been heterogeneous. Futibatinib, an irreversible FGFR1–4 inhibitor, has been evaluated in patients with gastric or gastroesophageal junction cancer harboring FGFR2 amplification [48]. Other inhibitors, including pemigatinib and tasurgratinib, have contributed early-phase or selected clinical data [24,25]. These studies support the biological relevance of FGFR2-altered gastric cancer, but they also illustrate why FGFR targeting has historically been difficult in this disease. Potential limitations include the low prevalence of truly FGFR-dependent tumors, heterogeneous or subclonal FGFR2 amplification, non-equivalent biomarker definitions, coexisting oncogenic drivers and adaptive bypass signaling through parallel pathways.

These issues help explain why weak or inconsistent signals with pan-FGFR inhibition should not be interpreted in a simplistic way. They do not invalidate FGFR2b as an antibody-accessible target, but they do caution against assuming that all FGFR2-altered or FGFR2b-positive tumors share the same therapeutic vulnerability. Toxicity-related dose interruption or discontinuation may further limit sustained kinase inhibition, although modality-specific safety considerations are discussed separately in Section 8. Overall, the experience with small-molecule FGFR inhibitors reinforces the central message of this review: the clinically relevant biomarker layer depends on the therapeutic modality being used.

### 7.7. Antibody–Drug Conjugates and Other Emerging Modalities

Other FGFR2-directed approaches, including antibody–drug conjugates and novel biologics, have been explored as potential strategies in FGFR2-altered cancers. Early clinical development of the FGFR2 antibody–drug conjugate aprutumab ixadotin demonstrated the feasibility of payload-based FGFR2 targeting, but also illustrated the challenges of toxicity and therapeutic window in advanced solid tumors [49]. Although this agent is distinct from current FGFR2b-directed antibody therapy, it highlights an important principle: the therapeutic modality determines which aspects of FGFR2 biology matter most.

For antibody–drug conjugates, target density, internalization, payload sensitivity, and bystander effect may be as important as receptor signaling. For immune-engaging biologics, immune contexture and tumor accessibility may become central. For broader FGFR inhibition, pathway activation and kinase dependence are more relevant. These differences are important because resistance mechanisms will likely vary across modalities. Loss of surface antigen may be especially relevant for antibody-based treatment, kinase-domain resistance for small-molecule inhibitors, and payload resistance for antibody–drug conjugates.

These emerging approaches remain less established than bemarituzumab-based therapy in gastric cancer, but they are important for future sequencing. If FGFR2b-directed therapy becomes part of standard treatment, subsequent strategies may need to address tumors that retain FGFR2 pathway dependence, tumors that lose FGFR2b expression, and tumors that escape through parallel pathways. The development of new modalities should therefore be linked to resistance characterization from the outset. The main clinical studies and therapeutic strategies discussed in this section are summarized in Table 2, with emphasis on biomarker selection, treatment setting, study design and interpretive relevance rather than direct cross-trial comparison.

### 7.8. Interpretation of the Current Evidence

The current clinical evidence supports FGFR2b as an actionable target in a biomarker-selected subset of advanced gastric and gastroesophageal junction cancer. The FIGHT trial established clinical proof of concept, while phase III development is refining the magnitude of benefit, optimal biomarker threshold, safety profile, and place of FGFR2b-directed therapy within first-line treatment. Importantly, these studies should not be interpreted only as evaluations of a drug regimen. They also test whether FGFR2b protein expression can function as a reproducible and clinically meaningful selection tool across diverse clinical settings.

Several questions remain central for interpretation. How much FGFR2b expression is sufficient for therapeutic relevance? Does diffuse expression predict benefit more reliably than focal expression? Should FGFR2 amplification be used as a complementary biomarker in selected cases? How should FGFR2b-directed therapy be combined or sequenced with immune checkpoint inhibition, anti-angiogenic therapy, CLDN18.2-directed therapy, HER2-targeted therapy, or other emerging strategies? Which mechanisms of progression are most common after exposure to FGFR2b-directed therapy?

Thus, the clinical evidence does not close the biomarker discussion; it makes the need for precise FGFR2b interpretation more urgent. FGFR2b-directed therapy is clinically promising, but its ultimate value will depend on biomarker standardization, mature and context-specific clinical data, resistance characterization, and real-world implementation. As FGFR2b-directed therapy advances, toxicity and tolerability become part of biomarker interpretation, because the threshold for positivity must ultimately support a favorable benefit–risk balance.

## 8. Safety, Quality of Life and Biomarker Implementation

The clinical value of FGFR2b testing will ultimately depend on whether it helps identify patients for whom FGFR2b-directed therapy offers a favorable net benefit. This is especially relevant for a treatment strategy that is being developed mainly in combination with established systemic regimens, where added efficacy must be weighed against additional toxicity, treatment complexity, dose modifications, and patient-reported outcomes.

As discussed in previous sections, broad immunohistochemical definitions identify more patients, whereas stricter thresholds enrich for tumors with more extensive target expression. Safety considerations add another dimension to this threshold question. If treatment-related toxicity is clinically meaningful, the optimal cut-off should not be chosen only to maximize eligibility. It should ideally identify the population in which FGFR2b-directed therapy provides sufficient benefit to justify additional exposure. This is particularly relevant for tumors with focal, borderline, or heterogeneous FGFR2b staining, where target presence may be documented but therapeutic dependence may be less certain.

Ocular and corneal adverse events have been a characteristic safety consideration in the clinical development of bemarituzumab. In FIGHT, corneal events were more frequent in the bemarituzumab-containing arm than in the control arm and require appropriate monitoring and management when FGFR2b-directed therapy is used [9]. The final analysis continued to support the clinical activity of bemarituzumab plus mFOLFOX6 while maintaining ocular safety as a relevant aspect of treatment delivery [10]. These events are clinically important because they may affect symptoms, dose modification, treatment duration, and quality of life.

The safety question becomes more complex in combination regimens. Bemarituzumab has been evaluated with mFOLFOX6 in the first-line setting, while later-line and investigational strategies are assessing combinations with established chemotherapy, anti-angiogenic, or chemoimmunotherapy backbones. RAINBIRD trial is evaluating bemarituzumab with paclitaxel and ramucirumab in FGFR2b-positive advanced gastric or gastroesophageal junction cancer after intolerance or refractoriness to first-line fluoropyrimidine-based chemotherapy [47]. FORTITUDE-102 is evaluating bemarituzumab with mFOLFOX6 and nivolumab in previously untreated FGFR2b-overexpressing advanced disease [12]. These studies are important not only for efficacy, but also for defining whether FGFR2b-directed therapy can be integrated into multi-agent regimens without compromising tolerability.

Toxicity profiles should also be interpreted according to therapeutic modality. Small-molecule FGFR inhibitors differ from FGFR2b-directed antibodies because they inhibit FGFR kinase activity more broadly and are often used in genomically selected FGFR-altered tumors rather than FGFR2b IHC-defined disease. Hyperphosphatemia, mucosal toxicity, gastrointestinal symptoms, fatigue, skin or nail changes, and ocular events are among the adverse events associated with FGFR kinase inhibition. In gastric or gastroesophageal junction cancer specifically, futibatinib has been evaluated in patients with FGFR2 amplification, providing disease-relevant evidence for this broader FGFR-inhibitor approach [48]. Similarly, FGFR2-directed antibody–drug conjugates raise distinct safety questions related to payload biology, internalization, and therapeutic window. The first-in-human phase I study of aprutumab ixadotin illustrated that payload-based FGFR2 targeting is feasible, but also that tolerability can limit development [49].

Patient-reported outcomes add an important dimension to this discussion. In advanced gastric cancer, treatment value is not determined only by survival or radiologic response; preservation of quality of life is also central. In the FIGHT health-related quality-of-life analysis, bemarituzumab plus mFOLFOX6 was associated with sustained health-related quality of life relative to mFOLFOX6 alone during the evaluated period [46]. These data support the idea that biomarker-directed treatment intensification should be assessed not only through conventional efficacy and safety endpoints, but also through patient-centered outcomes.

Overall, safety and quality-of-life data should inform how FGFR2b positivity is translated into treatment decisions. The clinically useful threshold may be the one that best identifies patients with favorable net benefit, not simply the one that detects the largest positive population. This is particularly relevant as FGFR2b-directed therapy is evaluated across different combinations and treatment lines. Even when FGFR2b expression is sufficient and treatment is feasible, therapeutic pressure may reveal or select biological escape routes.

## 9. Resistance to FGFR2b-Targeted Therapy: A Five-Layer Model

**Figure 3:**
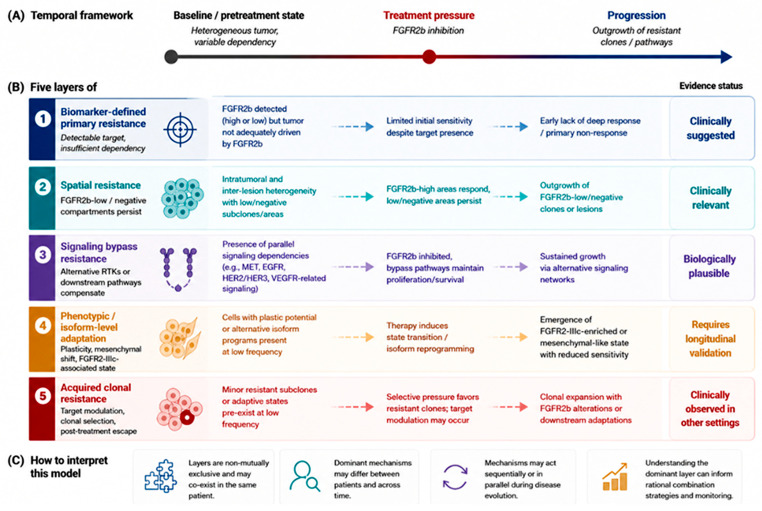
A five-layer model of resistance to FGFR2b-directed therapy. (**A**) Temporal framework illustrating how resistance may emerge from baseline tumor heterogeneity, treatment pressure and disease progression. (**B**) Five non-mutually exclusive resistance layers are proposed: biomarker-defined primary resistance, spatial resistance, signaling bypass, phenotypic or isoform-level adaptation, and acquired clonal resistance. (**C**) Interpretive principles emphasizing that these mechanisms may coexist, vary between patients and evolve over time, supporting resistance-informed monitoring and rational combination strategies (The five resistance layers shown here correspond to the evidence-graded framework summarized in Table 3 and should be interpreted according to the current level of empirical support for each mechanism; readers should refer to the corresponding sections of the main text for the evidence base, assay-specific limitations, and clinical interpretation underlying the relationships illustrated here).

Resistance to FGFR2b-directed therapy should be understood as a layered process rather than as a single molecular event. FGFR2b is simultaneously a protein biomarker, a receptor involved in oncogenic signaling, and a therapeutic target. Resistance may therefore arise from insufficient biomarker precision, uneven target distribution, activation of alternative pathways, phenotypic adaptation, or clonal evolution under treatment pressure. Some of these mechanisms are supported by direct gastric cancer data, whereas others remain biologically plausible and require validation in patients treated with FGFR2b-directed therapy. A layered model helps separate established observations from hypotheses that should be tested prospectively. The proposed resistance framework is summarized in Figure 3, which organizes potential mechanisms of escape according to timing, biological level and degree of clinical validation.

**Table 3:** Proposed resistance layers in FGFR2b-directed therapy and current level of evidence.

Resistance Layer	Main Mechanism	Examples	Current Level of Evidence	Practical Implication
Layer 1. Biomarker-defined primary resistance	FGFR2b is detectable but not biologically dominant	Low percentage of positive cells; borderline staining; FGFR2b expression without pathway dependence	Supported by gastric cancer biomarker and FIGHT subgroup data	Requires careful IHC thresholding and reporting of intensity, percentage and distribution
Layer 2. Spatial resistance	Uneven target distribution within or between lesions	FGFR2b-high and FGFR2b-low regions; discordant primary and metastatic sites; peritoneal sampling limitations	Supported by gastric cancer heterogeneity data; limited direct treatment-linked evidence	May explain mixed response and argues for representative tissue sampling or reassessment
Layer 3. Signaling bypass	Tumor survival shifts to parallel RTK or downstream pathways	MET, EGFR, HER2/HER3, VEGFR-related signaling; MAPK or PI3K–AKT reactivation	Biologically plausible in gastric cancer and supported by broader RTK resistance literature; limited direct evidence after bemarituzumab	Supports mechanism-driven molecular profiling at progression and rational combinations
Layer 4. Phenotypic or isoform-level adaptation	Tumor-cell plasticity reduces dependence on FGFR2b	EMT-like programs; FGFR2b-to-FGFR2c/IIIc shift; ligand-context changes	Supported by isoform biology and FGFR2c/mesenchymal associations; not yet established as acquired resistance to FGFR2b antibodies	Requires paired baseline/progression samples, RNA-based assays and spatial approaches
Layer 5. Acquired clonal resistance	Resistant clones expand or emerge under therapy	Loss/downregulation of FGFR2b; expansion of FGFR2b-low clones; acquired bypass alterations; downstream reactivation	Well established as a general targeted-therapy principle; incompletely characterized in bemarituzumab-treated gastric cancer	Requires serial tissue and ctDNA monitoring, with drug-class-specific interpretation

### 9.1. Layer 1: Biomarker-Defined Primary Resistance

Building on the distinction between FGFR2b expression and functional dependency discussed earlier, biomarker-defined primary resistance refers to tumors in which FGFR2b is detectable but not sufficiently dominant to determine therapeutic sensitivity. This may occur when expression is focal, borderline, or present in only a small fraction of tumor cells, or when other oncogenic drivers reduce reliance on FGFR2b signaling.

The FIGHT final analysis suggested that patients with FGFR2b overexpression in at least 10% of tumor cells appeared to derive greater benefit than the broader biomarker-selected population [10]. This observation supports the relevance of biomarker threshold, but it should be interpreted as one component of resistance biology rather than a complete predictor of outcome.

### 9.2. Layer 2: Spatial Resistance

As outlined in Section 6, FGFR2b expression may be spatially heterogeneous within and across tumor sites [22]. In the resistance model, the key implication is that FGFR2b-low or FGFR2b-negative compartments may persist despite treatment of FGFR2b-high disease. This can produce incomplete response, mixed response, or early progression in selected lesions.

Spatial resistance may therefore be present before therapy begins. Treatment does not necessarily create resistant disease de novo; it may reveal pre-existing compartments that were under-sampled or biologically less dependent on FGFR2b. This distinction is important because spatial resistance is not fully solved by improving systemic drug potency. It requires better sampling, more informative reporting, and, eventually, correlation between staining distribution and lesion-level response [12].

### 9.3. Layer 3: Signaling Bypass Resistance

A third layer involves pathway compensation. FGFR2b signaling converges on downstream networks such as MAPK and PI3K–AKT, which can also be activated by other receptor tyrosine kinases. If alternative pathways become dominant, FGFR2b blockade may be insufficient even when the receptor remains detectable.

Potential bypass routes include MET, EGFR, HER2/HER3, VEGFR-related signaling, and downstream pathway activation. These mechanisms are biologically plausible in gastric cancer, where receptor tyrosine kinase redundancy and co-alterations are well recognized. However, their exact frequency after FGFR2b-directed therapy remains incompletely characterized. Therefore, bypass signaling should be framed as a priority resistance category for translational study, not as a fully mapped clinical sequence [50].

This layer also provides the rationale for combination strategies. However, combinations should be mechanism-driven. The presence of a plausible bypass pathway does not automatically justify adding another agent unless there is evidence that the pathway contributes to resistance in a defined subgroup.

### 9.4. Layer 4: Phenotypic and Isoform-Level Adaptation

A fourth layer involves tumor-cell plasticity and FGFR2 isoform biology. As stated before, FGFR2b/IIIb is generally linked to epithelial biology, whereas FGFR2c/IIIc has been associated with more mesenchymal states. This raises the possibility that epithelial–mesenchymal transition-like programs, splicing changes, or isoform shifts could reduce sensitivity to FGFR2b-directed therapy. Recent data have highlighted the biological relevance of FGFR2-IIIc in gastric and gastroesophageal junction cancer, showing its association with mesenchymal transition and unfavorable tumor behavior. These findings support the plausibility of isoform-level adaptation as a resistance-relevant process. However, it would be premature to state that FGFR2b-to-FGFR2c switching is an established clinical mechanism of resistance after bemarituzumab. This hypothesis requires longitudinal validation using paired baseline and progression samples [23].

Phenotypic adaptation may also occur without complete loss of FGFR2b expression. Tumor cells may retain detectable receptor expression but become less dependent on it if survival programs shift toward alternative transcriptional states, stromal interactions, or downstream signaling routes.

### 9.5. Layer 5: Acquired Clonal Resistance

The final layer is acquired clonal resistance under treatment pressure. Possible mechanisms include reduction or loss of FGFR2b expression, expansion of FGFR2b-low clones, acquisition or enrichment of bypass alterations, and downstream pathway reactivation. These mechanisms may overlap with the earlier layers, but the distinguishing feature is timing: they become clinically relevant after exposure to FGFR2b-directed therapy.

Acquired resistance is not yet well characterized in gastric cancer treated with FGFR2b-directed agents. This creates an important opportunity for future studies. Progression biopsies, serial molecular profiling, and paired baseline–progression analyses could determine whether resistance is most often driven by antigen modulation, pathway bypass, isoform changes, or mixed mechanisms across metastatic sites [51].

Resistance mechanisms are also likely to differ by therapeutic modality. For FGFR2b-directed antibodies, antigen density and membrane expression may be central. For small-molecule FGFR inhibitors, kinase-domain alterations or pathway reactivation may be more relevant. For FGFR2-directed antibody–drug conjugates, internalization, payload sensitivity, and target density may become decisive. Therefore, the term “FGFR2 resistance” should always be interpreted in relation to the drug class being discussed [51].

Because the evidence supporting resistance to FGFR2b-directed therapy is uneven across mechanisms, the proposed resistance layers should be interpreted according to their current empirical support. Table 3 summarizes each layer, its main biological rationale, representative mechanisms, level of evidence and practical implication. This evidence-oriented framing is intended to distinguish mechanisms observed or strongly supported in gastric cancer from those that remain extrapolated from broader FGFR-driven or targeted-therapy models.

### 9.6. Clinical Implications of the Five-Layer Model

The five-layer model reframes resistance as a continuum from baseline biomarker uncertainty to treatment-induced tumor evolution. It suggests that FGFR2b-directed therapy may be limited by insufficient target coverage, spatially heterogeneous expression, competing survival pathways, phenotypic adaptation, and acquired clonal selection. These mechanisms are not mutually exclusive and may coexist in the same patient.

For clinical and translational studies, the key question should not be only whether FGFR2b is present at baseline, but whether it is sufficiently expressed, spatially distributed, biologically relevant, stable under treatment, and dominant relative to alternative escape pathways. These layers describe tumor-centered mechanisms of resistance, but they do not fully capture the context in which treatment occurs. FGFR2b-directed therapy is delivered within a complex tumor ecosystem, where stromal organization, vascular supply, immune composition and anatomical site may all influence therapeutic effect.

## 10. FGFR2b and the Tumor Microenvironment: Beyond Tumor-Cell Signaling

FGFR2b-directed therapy is usually framed around tumor-cell target expression, but treatment response is unlikely to depend only on receptor status within malignant cells. Gastric cancer develops within a complex tissue ecosystem that includes fibroblasts, endothelial cells, immune cells, extracellular matrix, soluble growth factors, and anatomical niches such as the peritoneal cavity. These components may influence how accessible the target is, how strongly tumor cells depend on FGFR2b signaling, and whether alternative survival cues are available when FGFR2b is inhibited. For this reason, the tumor microenvironment should be considered a potential modifier of FGFR2b-directed therapy rather than a separate background feature [52].

The role of the microenvironment is especially relevant because fibroblast growth factor signaling is inherently context-dependent. FGFR activity is shaped by ligand availability, receptor expression, stromal interactions, and downstream pathway crosstalk. Although FGFR2b is mainly discussed as an epithelial tumor-cell target, the broader FGF–FGFR network participates in epithelial–stromal communication, tissue repair, angiogenesis, and remodeling processes. Therefore, the clinical effect of FGFR2b-directed treatment may be influenced not only by receptor expression on tumor cells, but also by the surrounding tissue architecture in which those cells reside. One important microenvironmental dimension is stromal organization. Cancer-associated fibroblasts can alter extracellular matrix composition, increase tissue stiffness, and create physical or biochemical barriers to drug penetration. In gastric cancer, stromal-rich tumor regions may influence therapeutic accessibility even when the target antigen is present. This does not mean that FGFR2b-directed therapy is ineffective in stromal-rich disease, but it suggests that target expression and target reachability are not identical. A tumor region may be FGFR2b-positive by immunohistochemistry, yet still differ in drug exposure depending on stromal density, vascularization, and local tissue pressure [53].

Stromal cells may also provide compensatory growth and survival signals. If FGFR2b blockade reduces one tumor-cell signaling route, paracrine factors from fibroblasts, endothelial cells, macrophages, or other microenvironmental components may support persistence of residual disease. This idea is consistent with the broader principle, discussed in the resistance section, that pathway inhibition can be bypassed through alternative signaling. The microenvironment adds another layer to this process by supplying external cues rather than tumor-cell-autonomous alterations alone [53].

Angiogenesis and vascular function represent another relevant axis. FGFR signaling has biological links to vascular remodeling and angiogenic pathways, and advanced gastric cancer is frequently treated with anti-angiogenic therapy in later-line settings. The clinical evaluation of bemarituzumab with paclitaxel and ramucirumab in RAINBIRD trial reflects interest in integrating FGFR2b targeting with an established anti-angiogenic backbone, although the study should be interpreted as a clinical evaluation of combination therapy rather than proof of a specific microenvironmental mechanism [47]. From a translational perspective, such combinations raise important questions: does vascular modulation improve drug delivery, suppress compensatory angiogenic signaling, or benefit only selected microenvironmental states? These questions remain open.

The immune microenvironment may also influence FGFR2b-directed therapy. This is particularly relevant as FGFR2b targeting is being evaluated with chemotherapy and immune checkpoint inhibition in FORTITUDE-102 [12]. At present, the relationship between FGFR2b expression and immune responsiveness in gastric cancer is not fully defined. FGFR2b-positive tumors may vary in PD-L1 status, immune infiltration, microsatellite instability, and other immune-related features. Therefore, it would be premature to assume that FGFR2b positivity predicts sensitivity or resistance to immunotherapy. The more appropriate question is whether FGFR2b-directed therapy can be combined with immune checkpoint inhibition in a way that improves outcomes in a biomarker-defined population.

Peritoneal disease provides a clinically important example of how anatomical microenvironment may shape FGFR2b-directed therapy. As discussed earlier, FGFR2b expression can differ between primary tumors and peritoneal dissemination, and peritoneal lesions exist within a distinct biological compartment. Ascites, mesothelial interaction, stromal organization, hypoxia, and limited vascular access may influence both drug exposure and tumor behavior. In this setting, target positivity alone may not fully predict treatment effect. Future studies of FGFR2b-positive gastric cancer should therefore consider lesion site and pattern of spread when interpreting response and resistance [8,38,39].

A key translational challenge is that most biomarker testing currently focuses on tumor-cell expression, while microenvironmental variables are rarely incorporated into patient selection. Standard FGFR2b immunohistochemistry can identify the presence of the target, but it does not measure stromal density, immune exclusion, vascular accessibility, or paracrine signaling. This gap may partly explain why two tumors with similar FGFR2b staining could respond differently. One may be broadly target-expressing and accessible, whereas another may show comparable staining but exist within a microenvironment that limits drug penetration or supports alternative survival pathways [52].

The microenvironment also complicates interpretation of resistance. If progression occurs during FGFR2b-directed therapy, it may not always reflect loss of FGFR2b expression or a tumor-cell-intrinsic bypass alteration. In some cases, resistant disease may be supported by stromal remodeling, vascular adaptation, immune escape, or anatomical sanctuary sites. These possibilities should be explored carefully rather than assumed. Paired tissue analysis, spatial profiling, multiplex immunohistochemistry, and correlation of lesion-level response with microenvironmental features may help determine whether microenvironmental factors contribute to treatment outcome.

From a therapeutic perspective, the microenvironment provides both challenges and opportunities. It may limit FGFR2b-directed therapy by reducing target access or providing compensatory signals, but it may also offer rational combination strategies. Anti-angiogenic therapy, immune checkpoint inhibition, chemotherapy, or future stromal-modulating approaches could theoretically complement FGFR2b targeting in selected contexts. However, such combinations should be guided by clinical evidence and biomarker rationale. The existence of a plausible microenvironmental mechanism is not sufficient by itself to justify treatment intensification.

Overall, FGFR2b should be interpreted as a tumor-cell biomarker operating within a broader tissue ecosystem. Its therapeutic relevance may depend on the interaction between receptor expression, pathway dependence, spatial distribution, vascular access, stromal structure, immune contexture, and anatomical site. This perspective does not weaken the value of FGFR2b as a target; rather, it helps explain why target positivity alone may be insufficient to predict the full clinical behavior of FGFR2b-positive disease. In clinical practice, therefore, FGFR2b is unlikely to function as an isolated decision point; its value will depend on how it is interpreted within the expanding biomarker landscape of gastric cancer.

## 11. FGFR2b in the Multi-Biomarker Ecosystem of Gastric Cancer

The clinical relevance of FGFR2b cannot be defined in isolation. Gastric and gastroesophageal junction cancers are increasingly evaluated through a multi-biomarker framework that includes HER2, PD-L1 combined positive score, mismatch repair or microsatellite instability status, CLDN18.2, and, in selected cases, broader genomic profiling. Within this landscape, FGFR2b should be interpreted not merely as an additional positive-or-negative result, but as one component of a wider therapeutic decision.

This issue is increasingly important because several actionable or potentially actionable biomarkers may coexist in the same tumor. A patient may have a HER2-negative but FGFR2b-positive tumor, a tumor with both FGFR2b and CLDN18.2 expression, an FGFR2b-positive tumor with high PD-L1 expression, or FGFR2b positivity accompanied by other receptor tyrosine kinase alterations. These scenarios require a clinical hierarchy: which target is most actionable in a given line of therapy, which biomarker has the strongest evidence, and whether the available data support combination, sequencing, or prioritization of one strategy over another.

### 11.1. FGFR2b and HER2

HER2 remains the most established receptor tyrosine kinase biomarker in gastric and gastroesophageal junction cancer [5]. FGFR2b, by contrast, is an emerging therapeutic target whose most advanced clinical development has focused largely on HER2-negative disease. This distinction is clinically important because FGFR2b-directed therapy has been positioned mainly as a strategy for patients whose tumors do not qualify for HER2-directed treatment within current trial designs [11,12].

The relationship between HER2 and FGFR2b should nevertheless be interpreted with some flexibility. Although the main confirmatory FGFR2b-directed studies have focused on HER2-negative tumors, gastric cancer may show complex receptor tyrosine kinase biology, and rare co-positive or spatially heterogeneous cases might occur. For such cases, the clinically relevant question is not simply whether both markers are detectable, but whether one target is dominant, more homogeneously expressed, or supported by stronger evidence in the intended treatment line.

Therefore, HER2 status will likely remain an early decision point in treatment selection, while FGFR2b testing may add value particularly among HER2-negative tumors. Future studies should clarify whether HER2/FGFR2b co-positive cases represent a rare overlap, spatially distinct subclones, or a biologically meaningful subgroup requiring specific sequencing strategies.

### 11.2. FGFR2b and CLDN18.2

The relationship between FGFR2b and CLDN18.2 is especially important because both are protein biomarkers assessed primarily by immunohistochemistry and both are linked to antibody-based treatment strategies. They therefore share practical challenges related to staining thresholds, distribution of expression, tissue sampling, and interpretation of borderline cases. These methodological issues have already been discussed for FGFR2b in earlier sections; here, the key point is that co-expression may create therapeutic prioritization questions.

Recent tissue-based data indicate that FGFR2b and CLDN18.2 expression can overlap in a subset of gastric cancers. In a large tissue microarray study of 1538 gastric carcinomas, Ahn et al. evaluated the correlation and overlap between CLDN18.2 and FGFR2b overexpression, highlighting the need to understand how these markers coexist in the same disease space [22]. However, the presence of overlap does not yet establish a treatment rule. It remains unclear whether dual-positive tumors should be treated preferentially with one target-directed strategy, whether sequencing should depend on expression level and line of therapy, or whether combination approaches may eventually be justified.

For now, FGFR2b/CLDN18.2 co-expression should be viewed as an important clinical and translational question rather than as a resolved algorithm. Future studies should evaluate whether the two antigens are expressed in the same tumor regions or in different compartments, whether one is more stable under treatment pressure, and whether dual-positive tumors have distinct outcomes. Until such data are available, co-positivity should prompt integrated interpretation rather than automatic treatment intensification.

### 11.3. FGFR2b and Immune Biomarkers

Immune biomarkers add a different type of decision layer. PD-L1 combined positive score, mismatch repair deficiency or microsatellite instability, and tumor mutational burden may influence the use of immune checkpoint inhibition in gastric cancer. FGFR2b status does not replace these markers and should not be treated as a surrogate for immune responsiveness [2,3,4].

The clinically relevant question is whether FGFR2b-directed therapy can be integrated with immune checkpoint inhibition in appropriately selected patients. At present, FGFR2b positivity alone should not be used to infer sensitivity or resistance to immunotherapy. The more appropriate approach is to interpret FGFR2b alongside PD-L1 CPS, MSI/MMR status, clinical context, and available trial evidence. This avoids turning FGFR2b into an immune biomarker before supporting data exist.

### 11.4. FGFR2b and Other Receptor Tyrosine Kinase Alterations

As discussed in the resistance model, receptor tyrosine kinase co-alterations may influence dependence on FGFR2b and may provide alternative signaling routes. In this chapter, the practical implication is biomarker prioritization. If a tumor is FGFR2b-positive but also harbors another potentially relevant receptor tyrosine kinase alteration, such as MET amplification or strong activation of another pathway, clinicians and trial designers must consider whether FGFR2b is likely to be the dominant therapeutic vulnerability.

This does not mean that all FGFR2b-positive tumors require broad combination therapy. Rather, it supports the value of broader molecular profiling in selected situations: discordant biomarker results, unexpectedly aggressive disease, mixed response, or progression after targeted treatment. Genomic profiling may help identify co-alterations that influence treatment choice or trial eligibility, while FGFR2b immunohistochemistry remains essential for identifying the protein target of FGFR2b-directed antibody therapy.

The terminology is important. As mentioned before, a tumor described as “FGFR2b-positive” is not necessarily the same as a tumor whose growth is exclusively FGFR2-driven. When additional receptor tyrosine kinase alterations are present, FGFR2b should be interpreted as part of a network of potential dependencies rather than as an isolated driver.

### 11.5. FGFR2b and the Emerging Antigen-Targeted Landscape

The expansion of antibody-based and antibody–drug conjugate strategies in gastric cancer is changing how protein biomarkers are interpreted. HER2, CLDN18.2 and other antigen targets are increasingly relevant, and FGFR2b may eventually need to be considered within this broader antigen-targeted landscape.

This matters because antigen-directed therapies share some interpretive challenges. Target density, distribution, membrane localization, internalization and persistence after prior therapy may all influence therapeutic relevance. These principles were discussed earlier for FGFR2b heterogeneity and resistance; in the multi-biomarker context, the key issue is comparative prioritization. If several antigen targets are present, the most appropriate treatment may depend on which marker is more broadly expressed, which agent has stronger evidence in that treatment line, and which prior therapies may have altered antigen expression. At present, the evidence base for FGFR2b-directed antibody therapy is more advanced than for many hypothetical FGFR2b-directed ADC strategies in gastric cancer [11,12]. Therefore, discussion of the antigen-targeted landscape should remain forward-looking and should avoid implying that all antigen targets are equally actionable today.

### 11.6. Practical Sequencing Scenarios

The practical challenge is to translate multiple biomarker results into a treatment sequence. In a HER2-negative, FGFR2b-positive tumor with no other dominant actionable marker, FGFR2b-directed therapy may represent a logical biomarker-driven strategy if supported by the treatment setting and available evidence. In a tumor that is both FGFR2b-positive and CLDN18.2-positive, treatment choice may depend on line of therapy, expression level, antigen distribution, regulatory context, toxicity profile and access to therapy. In a tumor with high PD-L1 CPS or MSI-high status, immune-based therapy may remain central, while the role of adding or sequencing FGFR2b-directed therapy should be guided by clinical data rather than assumption.

These scenarios illustrate why multi-biomarker interpretation is becoming more important than single-marker detection. A useful clinical report may eventually need to present HER2, PD-L1 CPS, MSI/MMR, CLDN18.2, FGFR2b and selected genomic alterations in a coordinated way, with attention to tissue source and assay limitations. The goal is not only to identify every positive marker, but to determine which marker is therapeutically relevant at a particular decision point. Because FGFR2b will be implemented in an increasingly crowded biomarker landscape, its clinical value should be interpreted alongside established and emerging gastric cancer biomarkers. Table 4 summarizes practical differences in assay type, eligible population, heterogeneity, therapeutic strategy, predictive value, resistance patterns and clinical status.

This comparison highlights that FGFR2b shares key challenges with other protein biomarkers, particularly cut-off dependence and spatial heterogeneity, while also raising distinct questions because FGFR2b protein expression, FGFR2 amplification and FGFR pathway dependence are not interchangeable.

## 12. From Static Testing to Dynamic Implementation: A Practical Agenda for FGFR2b

The central question for the next phase of FGFR2b research is no longer whether FGFR2b can be detected in gastric and gastroesophageal junction cancer. The more clinically relevant question is how this biomarker should be implemented so that testing identifies patients most likely to benefit from FGFR2b-directed therapy, while avoiding overinterpretation of limited or ambiguous results. In this sense, FGFR2b is entering a transition from biomarker discovery to biomarker governance: definition, reporting, reassessment and clinical prioritization must become more standardized.

A first priority is to improve the quality and interpretability of baseline reporting. As mentioned, FGFR2b immunohistochemistry should not be reduced to a simple positive-or-negative label when the staining pattern is focal, borderline or heterogeneous. Reports should ideally include the percentage of tumor cells with clinically relevant membranous staining, staining intensity, specimen source, and a brief comment on whether expression appears diffuse or restricted. Such reporting would not require a new biomarker concept, but it would make the existing result more clinically useful. It would also allow future studies to correlate not only positivity, but also expression pattern, with treatment benefit.

From an operational pathology perspective, this requires attention to both pre-analytical and analytical variables. Pre-analytical factors include fixation time, tissue processing, the age of archival material, tumor cellularity and adequacy of biopsy material. These issues are particularly relevant in gastric cancer, where small endoscopic biopsies or limited metastatic samples may underrepresent heterogeneous membranous staining. Analytical variables are equally important. Antibody clone, staining platform, antigen retrieval protocol and scoring algorithm may all influence apparent FGFR2b positivity, and results should not be assumed to be interchangeable across assays unless formal validation has been performed.

Reporting should ideally include staining intensity, percentage of positive tumor cells, membranous localization, specimen type, anatomical site and any limitations related to tissue adequacy or spatial heterogeneity. Interobserver reproducibility should be assessed during assay implementation, particularly for borderline cases with focal or weak-to-moderate staining. Digital pathology may help standardize estimation of positive tumor-cell percentage, quantify spatial heterogeneity and support audit of difficult cases, but it should complement expert pathologist interpretation until clinically validated thresholds are established. External quality assessment programs and inter-laboratory validation will be important to ensure that FGFR2b testing remains reproducible across institutions, especially if treatment eligibility depends on narrow IHC cut-offs. These operational issues directly influence the apparent prevalence of FGFR2b positivity, the classification of discordant IHC/FISH or IHC/NGS cases and the clinical confidence with which FGFR2b-directed therapy can be recommended.

A second priority is to define when repeat assessment is informative and which modality should be used. Routine re-biopsy for every patient is unrealistic, but selected reassessment at progression could be valuable after exposure to FGFR2b-directed therapy, especially in cases with mixed response, unexpected early progression, peritoneal-dominant disease or planned enrolment in another targeted trial. The purpose of reassessment should be specific: to determine whether FGFR2b expression is retained, whether a progressing lesion differs from the baseline sample, whether FGFR2 amplification persists or is lost, or whether another actionable alteration has become clinically relevant. In this setting, ctDNA can complement tissue reassessment by tracking FGFR2 copy-number changes and emerging co-alterations over time. It may be particularly useful when metastatic tissue is inaccessible, when peritoneal disease is difficult to biopsy, or when a single tissue sample is unlikely to represent the full disease burden. Nevertheless, ctDNA should not be regarded as a full substitute for tissue-based FGFR2b assessment, because it cannot evaluate membranous protein expression, staining intensity, tumor-cell percentage or spatial distribution. A practical monitoring strategy may therefore require combined interpretation of baseline IHC, tissue or plasma NGS, radiologic response pattern and selective progression biopsy when feasible. Beyond routine tissue reassessment and ctDNA, translational studies should incorporate technologies capable of resolving heterogeneity at higher resolution. Serial biopsies can determine whether FGFR2b expression is retained or lost under treatment pressure, while single-cell sequencing may identify resistant subpopulations, coexisting lineage states and RTK-bypass programs that are obscured in bulk assays. Spatial transcriptomics and multiplex tissue imaging could further clarify whether FGFR2b-positive and FGFR2b-low compartments differ in stromal proximity, immune contexture, ligand availability or downstream pathway activation. In parallel, AI-assisted digital pathology may help quantify staining percentage, intensity and spatial distribution more reproducibly, provided that algorithms are trained and validated against expert pathology assessment. Together, these approaches could move FGFR2b interpretation from a static baseline label toward a longitudinal and spatially informed biomarker model.

A third priority is to make future trials biomarker-informative by design. Studies of FGFR2b-directed therapy should prospectively capture expression level, staining distribution, specimen type, co-biomarker status and, when feasible, paired progression samples. This would allow the field to move beyond simple eligibility criteria and toward clinically useful predictors of benefit. In particular, trials should clarify whether diffuse expression performs differently from focal expression, whether FGFR2 amplification adds predictive value to FGFR2b immunohistochemistry, and whether co-expression with markers such as CLDN18.2 or high PD-L1 CPS affects optimal sequencing.

A fourth priority is to integrate FGFR2b into real-world diagnostic workflows. Gastric cancer tissue is often limited, and multiple biomarkers may need to be assessed from small biopsies. FGFR2b testing should therefore be coordinated with HER2, PD-L1 CPS, MSI/MMR, CLDN18.2 and molecular profiling rather than requested as an isolated late-stage add-on. This is not only a logistical issue. Poor tissue stewardship can lead to incomplete biomarker assessment, delayed treatment decisions, or reliance on archival material that may not reflect current disease biology.

Finally, FGFR2b implementation will require a shift in how biomarker value is judged. The goal should not be to identify the largest possible FGFR2b-positive population, but to define the population in which the biomarker is reliable, therapeutically meaningful and clinically actionable. This requires linking the test result to efficacy, safety, quality of life, resistance patterns and treatment sequencing. A practical FGFR2b framework should therefore ask five questions: Is the target sufficiently expressed? Is the tested sample representative? Is FGFR2b likely to be a relevant dependency? Are there competing or coexisting biomarkers that change treatment priority? And is there a plan to reassess the disease if resistance emerges?

In this form, FGFR2b becomes more than an emerging target. It becomes a model for how new protein biomarkers should be implemented in gastric cancer: not as static labels, but as context-dependent variables that require standardized reporting, rational integration and selective longitudinal reassessment. This approach may help ensure that FGFR2b-directed therapy is developed with the same precision that the biomarker itself demands.

## 13. Conclusions

FGFR2b is emerging as a clinically relevant target in gastric and gastroesophageal junction cancer, but its importance lies not only in the availability of FGFR2b-directed therapy. It also illustrates a broader challenge in precision oncology: a biomarker may be actionable, yet still difficult to interpret if its expression is heterogeneous, context-dependent and influenced by therapeutic pressure.

The key lesson from FGFR2b-positive disease is that target detection and therapeutic meaning are not identical. FGFR2b expression can identify a potentially treatable subgroup, but clinical interpretation requires attention to the quality of the assay, the extent and distribution of expression, the biological context of the tumor, and the presence of other actionable markers. In this sense, FGFR2b should be viewed less as a static label and more as a biomarker whose relevance depends on how convincingly it defines a treatment-sensitive state.

The future of FGFR2b-directed therapy will therefore depend on the integration of three elements: robust testing, biologically informed patient selection, and resistance-aware clinical development. If these elements are aligned, FGFR2b has the potential to become more than an emerging target; it may serve as a model for how new protein biomarkers are implemented in gastric cancer, where therapeutic decisions increasingly require interpretation across multiple targets, tumor sites and lines of therapy.

## Figures and Tables

**Table 1:** FGFR2b protein expression and *FGFR2* amplification as partially overlapping biomarker layers in gastric/GEJ cancer.

Biomarker Layer	Main Assay	Representative Data	Concordance/Discordance Issue	Most Relevant Therapeutic Context	Practical Interpretation
FGFR2b protein expression	IHC	FORTITUDE-101 prescreening: 37.8% with any 2+/3+ staining; 16.2% with ≥10% 2+/3+ staining [13]	Broader IHC cut-offs identify more tumors than stringent cut-offs and may include amplification-negative cases	FGFR2b-directed antibodies	Best marker of antibody-accessible membranous target, but not a direct measure of pathway dependence
*FGFR2* amplification	FISH or tissue NGS	Generally, less frequent than broad FGFR2b IHC positivity; commonly reported in a small subset of gastric cancers	Amplified tumors may show variable FGFR2b protein expression depending on isoform, sampling and heterogeneity	FGFR kinase inhibitors; complementary evidence for FGFR2-driven biology	Supports genomic pathway activation, but does not prove FGFR2b surface expression
ctDNA *FGFR2* amplification	Plasma NGS	Can detect *FGFR2* amplification and co-alterations when tissue is unavailable or difficult to biopsy	Sensitivity may be reduced with low tumor shedding, especially in some peritoneal-dominant cases	Longitudinal monitoring; resistance assessment	Useful complement to tissue testing, but cannot assess FGFR2b protein expression or spatial distribution
FGFR2/FGFR2b transcript activity	RNA-based assays	Prevalence estimates are not standardized for routine selection	RNA-high tumors may not be protein-high; isoform-specific assays are needed to distinguish FGFR2b from FGFR2c	Translational research; isoform biology	Helpful for epithelial/mesenchymal state and isoform switching, but not yet routine-ready
Functional FGFR dependence	Phospho-protein, organoid, pathway or ex vivo assays	No established clinical prevalence	May diverge from both IHC and amplification because co-drivers can dominate signaling	Experimental selection for FGFR-directed therapy or combinations	Closest to biological addiction, but currently investigational
Composite biomarker model	IHC + FISH/NGS ± ctDNA/RNA	Not yet prospectively validated	May resolve IHC-positive/amplification-negative and amplification-positive/FGFR2b-low cases	Future clinical trials and resistance monitoring	May outperform single-assay selection, but needs validated algorithms

**Table 2:** Selected clinical studies of FGFR2/FGFR2b-directed therapeutic strategies in gastric and gastroesophageal junction cancer. Abbreviations: FGFR, fibroblast growth factor receptor; GEJ, gastroesophageal junction; HRQoL, health-related quality of life; IHC, immunohistochemistry. For ongoing phase III studies, the table summarizes study design and clinical rationale based on registry information rather than mature peer-reviewed efficacy datasets.

Study/Strategy	Therapeutic Approach	Biomarker Selection	FGFR2b IHC Cut-Off/Biomarker Definition	Setting	Design	Main Interpretive Value
FIGHT [9]	Bemarituzumab + mFOLFOX6 vs. placebo + mFOLFOX6	FGFR2b-selected disease; HER2-negative disease	FGFR2b IHC-defined positivity and/or FGFR2 amplification according to trial eligibility criteria	First-line advanced gastric/GEJ adenocarcinoma	Randomized, double-blind, placebo-controlled phase II	Established clinical proof of concept for FGFR2b-selected therapy in advanced gastric/GEJ cancer
FIGHT final analysis [10]	Bemarituzumab + mFOLFOX6 vs. placebo + mFOLFOX6	FGFR2b-positive disease; HER2-negative disease	FGFR2b IHC-positive disease; analysis highlighted the ≥10% FGFR2b-overexpression subgroup	First-line locally advanced or metastatic gastric/GEJ adenocarcinoma	Final analysis of randomized phase II trial	Reinforced clinical activity and supported further evaluation of expression-level effects, including the ≥10% FGFR2b-overexpression subgroup
FIGHT East Asian subgroup [45]	Bemarituzumab + mFOLFOX6 vs. placebo + mFOLFOX6	FGFR2b-overexpressing disease; HER2-negative disease	Same parent FIGHT biomarker framework; FGFR2b IHC-defined overexpression	First-line locally advanced or metastatic gastric/GEJ adenocarcinoma	Subgroup analysis of global phase II FIGHT trial	Provided regional subgroup data supporting continued evaluation of FGFR2b-directed therapy in East Asian patients
FIGHT HRQoL analysis [46]	Bemarituzumab + mFOLFOX6 vs. placebo + mFOLFOX6	FGFR2b-positive/FGFR2b-overexpressing disease	Same parent FIGHT biomarker framework; FGFR2b IHC-defined positivity/overexpression	First-line advanced gastric/GEJ adenocarcinoma	Health-related quality-of-life analysis from FIGHT	Suggested sustained health-related quality of life with bemarituzumab plus mFOLFOX6 relative to mFOLFOX6 alone
FORTITUDE-101 [11]	Bemarituzumab + mFOLFOX6 vs. placebo + mFOLFOX6	FGFR2b overexpression; HER2-negative disease	FGFR2b IHC-defined overexpression; prescreening analyses show cut-off dependence, including any 2+/3+ staining versus ≥10% 2+/3+ staining	Previously untreated unresectable locally advanced or metastatic gastric/GEJ adenocarcinoma	Randomized, double-blind, placebo-controlled phase III	Confirmatory first-line evaluation of FGFR2b-directed therapy with chemotherapy
FORTITUDE-102 [12]	Bemarituzumab + mFOLFOX6 + nivolumab vs. placebo + mFOLFOX6 + nivolumab	FGFR2b overexpression	FGFR2b IHC-defined overexpression according to trial eligibility criteria	Previously untreated unresectable locally advanced or metastatic gastric/GEJ adenocarcinoma	Phase Ib/III	Evaluates integration of FGFR2b-directed therapy into a chemoimmunotherapy backbone
RAINBIRD/WJOG18524G [47]	Bemarituzumab + paclitaxel + ramucirumab	FGFR2b-positive disease	FGFR2b IHC-defined positivity according to study criteria	Unresectable advanced/recurrent gastric or GEJ adenocarcinoma after intolerance or refractoriness to first-line fluoropyrimidine-based chemotherapy	Single-arm, multicenter phase II	Explores FGFR2b-directed therapy with an established later-line paclitaxel/ramucirumab backbone
Futibatinib phase II [48]	Futibatinib, irreversible FGFR1–4 inhibitor	FGFR2 amplification	Not FGFR2b IHC-based; genomic FGFR2 amplification	Advanced gastric/GEJ cancer harboring FGFR2 amplification	Open-label phase II	Evaluates broader FGFR pathway inhibition in a genomically selected population, distinct from FGFR2b IHC-based antibody selection
Pemigatinib/FiGhTeR trial [24]	Pemigatinib, FGFR inhibitor	Advanced/metastatic gastric or GEJ adenocarcinoma after trastuzumab-containing first-line therapy; biologic rationale related to FGFR pathway activation	Not primarily FGFR2b IHC-based; selection/rationale related to FGFR pathway activation rather than FGFR2b antibody-accessible protein expression	Second-line strategy after trastuzumab-containing therapy	Single-arm, open-label phase II	Illustrates FGFR inhibition in a selected second-line gastric/GEJ cancer setting, but should not be presented as established FGFR2b-directed therapy
Tasurgratinib phase I expansion [25]	Tasurgratinib, FGFR inhibitor	FGFR-altered cholangiocarcinoma or gastric cancer cohorts	Not FGFR2b IHC-based; FGFR alteration-based development program	Cholangiocarcinoma or gastric cancer	Expansion part of first-in-human phase I study	Provides early evidence for FGFR inhibition in gastric cancer within a broader FGFR-altered development program
Aprutumab ixadotin [49]	FGFR2-directed antibody–drug conjugate	Advanced solid tumors known to express FGFR2	FGFR2 expression-based selection; not directly equivalent to bemarituzumab FGFR2b IHC selection	Advanced solid tumors, including tumor types with FGFR2 expression	First-in-human phase I	Demonstrated feasibility of FGFR2-directed payload-based therapy, while highlighting modality-specific development constraints

**Table 4:** FGFR2b within the biomarker ecosystem of gastric and gastroesophageal junction cancer.

Biomarker	Main Assay	Eligible Population/Prevalence	Heterogeneity	Therapeutic Strategy	Predictive Value	Resistance/Escape	Clinical Status
FGFR2b	IHC; FISH/NGS or ctDNA for FGFR2 amplification	Cut-off dependent; 37.8% with any 2+/3+ and 16.2% with ≥10% 2+/3+ in FORTITUDE-101 prescreening	Intratumoral, inter-lesion and peritoneal discordance	FGFR2b antibody therapy; selected FGFR2-altered approaches	Emerging; strongest for membranous FGFR2b expression	FGFR2b-low clones, target loss, amplification heterogeneity, bypass RTKs, EMT/isoform adaptation	Late-stage development; not universal routine standard
HER2	IHC; ISH for equivocal cases	About 10–20%, variable by site and histology	Intratumoral and primary-metastatic discordance	HER2 antibodies and ADCs	Established for HER2-directed therapy	HER2 loss, heterogeneous expression, bypass signaling, downstream activation	Established clinical biomarker
CLDN18.2	IHC	Clinically relevant subset; prevalence varies by assay and cut-off	Heterogeneous target expression	CLDN18.2 antibody therapy and emerging modalities	Established for zolbetuximab in eligible HER2-negative disease	Antigen loss/heterogeneity, limited target coverage, microenvironmental barriers	Approved in selected jurisdictions
PD-L1/MSI–MMR	PD-L1 CPS IHC; MMR IHC, MSI PCR/NGS	PD-L1 depends on CPS cut-off; MSI-H/dMMR uncommon but important	PD-L1 heterogeneous; MSI/MMR more stable	Immune checkpoint inhibition	CPS threshold-dependent; MSI-H/dMMR strongly predictive	Immune exclusion, antigen-presentation loss, JAK/STAT alterations, suppressive stroma	Established component of treatment selection
Other actionable alterations	NGS, FISH or IHC depending on target	Individually uncommon	Often subclonal or site-specific	MET, EGFR, NTRK, RET and trial-based targets	Alteration-specific; often investigational	Polyclonal resistance, bypass signaling, downstream reactivation	Mostly trial-based or selected-use

## Data Availability

No new data were created in this study. Data sharing is not applicable to this article.
